# Icono: a universal language that shows what it says

**DOI:** 10.3389/fpsyg.2023.1149381

**Published:** 2023-07-28

**Authors:** Peter Kramer

**Affiliations:** Department of General Psychology, University of Padua, Padua, Italy

**Keywords:** icon, pictogram, Esperanto, auxiliary language, dyslexia, aphasia, cerebral palsy, autism

## Abstract

This article lays out the foundation of a new language for easier written communication that is inherently reader-friendly and inherently international. Words usually consist of strings of sounds or squiggles whose meanings are merely a convention. In *Icono*, instead, they typically are strings of icons that illustrate what they stand for. “Train,” for example, is expressed with the icon of a train, “future” with the icon of a clock surrounded by a clockwise arrow, and “mammal” with the icons of a cow and a mouse—their combination’s meaning given by what they have in common. Moreover, Icono reveals sentence structure graphically before, rather than linguistically after, one begins reading. On smartphones and computers, writing icons can now be faster than writing alphabetic words. And using simple pictures as words helps those who struggle with conditions like dyslexia, aphasia, cerebral palsy, and autism with speech impairment. Because learning its pronunciation or phonetic spelling is optional rather than a prerequisite, and because it shows what it says, Icono is bound to be easier to learn to read—and then easier to read—than any other language, including our own.

A picture is worth a thousand words. —International adage.

## 1. Introduction: stepping back to jump forward

### 1.1. Accommodating the writer

Good writers put readers first, it is said. The evolution of writing, however, headed in the opposite direction (Fischer, 2001; Coulmas, 2003). It started off quite well by letting little pictures and tally marks become carriers of meaning. These were either *pictograms* that illustrated the concrete object, quantity, or action they referred to, or *ideograms* that visualized the abstract idea that corresponded to their meaning. Over time, however, these hieroglyphs were exploited increasingly often for their pronunciation rather than their meaning. As hieroglyphs became less important as carriers of meaning, people cut corners drawing them, and they were simplified, reoriented, and transformed beyond recognition (see also Garrod et al., 2007). Eventually they morphed into letters—carriers of pronunciation only. Letters are a lot easier to write than hieroglyphs are to draw, a small set of them is enough, and reading the pronunciation of familiar words makes us recall their meaning. Considering all these advantages, alphabets and suchlike have been hailed as the pinnacle of the history of writing, or at least something close to it (Fischer, 2001; Coulmas, 2003; Torrez et al., 2019; Amit et al., 2022; Morin, 2022).

The last, but only partial, holdout against this presumed progress is Mandarin Chinese.^1^ Chinese script consists of *characters* composed of combinations of *radicals*—pictographic or ideographic root-words. Although fascinating, rightfully lauded for their beauty, and the main inspiration for the present article, these radicals and characters do not illustrate their meaning as intuitively as they could. Among the most intuitive are 口, 人, and 木, which represent “mouth,” “person,” and “tree,” and none of them very much resemble the thing they represent. This is a pity, because the worse a character illustrates its meaning, the harder it is to learn (Soemer and Schwan, 2012; see also Bellezza, 1996).

Most Chinese characters carry only a very broad hint to their meaning and—in compensation for this lack of precision—an additional, presumed, but rather unreliable, hint to their pronunciation (Sampson, 1985; Coulmas, 2003; Morin, 2022). Confusingly, even this dubious hint to pronunciation is ultimately composed of radicals, which illustrate their own meaning and not their own pronunciation. Worse, typically the hints to the meaning and the pronunciation of complex characters are themselves simpler characters in their own right with, respectively, either an unrelated pronunciation or an unrelated meaning—a source of confusion for even the most fluent reader (see Section 1.3; O’Seaghdha and Marin, 1997; Zhou and Marslen-Wilson, 1999a,b; Yeh et al., 2017).

Besides Chinese, several other languages have adopted Chinese characters. Eventually, though, these characters were either augmented (Japanese) or completely replaced (Vietnamese and, for the most part, also Korean) with phonetic tokens. Even in China itself, to combat illiteracy, former chairman Mao Zedong pushed for the replacement of Chinese script with *Pinyin*—“spell sound,” an alphabetic version of Chinese (Fischer, 2001; Mullaney, 2017). In the end, the Chinese kept their characters, but in mainland China, they were simplified.^2^

Recently, in a far-reaching twist of fate, Pinyin has become quite popular after all, but not as a replacement of Chinese characters but as input for smartphone apps and word processors that instantly convert Pinyin into either simplified or traditional Chinese characters (Mullaney, 2017, 2021). Using Pinyin, as explained later, one can now write Chinese characters faster than one can spell out alphabetic words. And interestingly, with the same speed and ease, Pinyin can now also be used to write modern emojis.

### 1.2. Accommodating the reader

Now that technology allows us to separate writing’s input (e.g., Pinyin) from its output (e.g., Chinese characters or emojis), the question arises which output serves readers best: one that expresses the pronunciation of words or one that offers hints to their meaning. The sounds of words like dripple, giggle, or clap remind one of their meaning and people learn and understand such “onomatopoeic” words faster than other ones (Perniss et al., 2010; Lupyan and Winter, 2018; Röders et al., 2022). Far more often, however, the pronunciation of words does not, on its own, help bring their meanings to mind (Perniss et al., 2010; Lupyan and Winter, 2018). It does so only if one has previously learned the rather arbitrary, and thus nonintuitive, association between the two. If words consist of icons, however, then as long as these icons offer at least a slight hint to word meaning, they are already more helpful to readers than are letters.

**Figure 1:**
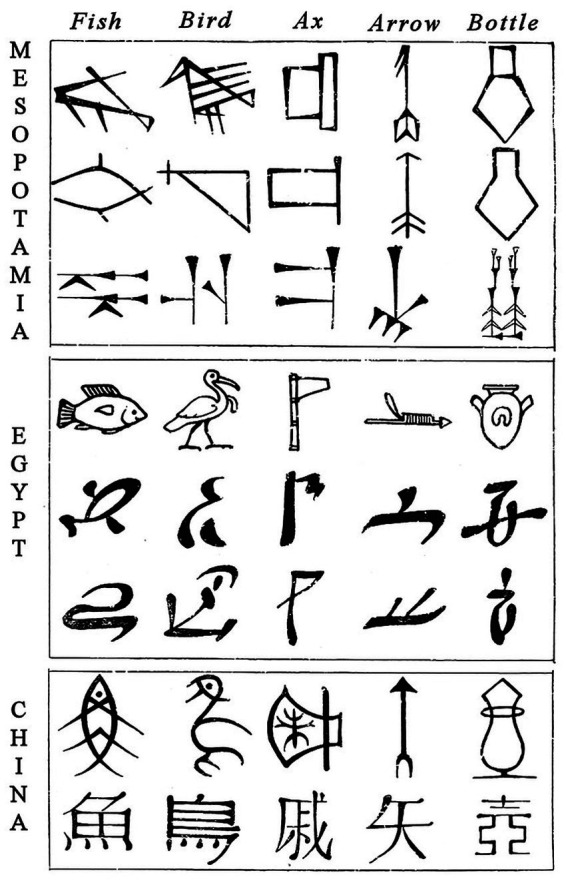
Mesopotamian, Egyptian, and Chinese hieroglyphs (Maspero, 1916; image in the public domain: https://archive.org/). Within each panel, the top row represents early, more concrete hieroglyphs, and subsequent rows later, more abstract versions of them. The Egyptian hieroglyphs eventually evolved into our present-day letters (Fischer, 2001; Coulmas, 2003; Nawar, 2020).

Indeed, millennia-old pictograms of a fish and a bird are still easy to read, even if one does not know the language of the ancient scribes that wrote them (Figure 1). Yet, in the regions where these pictograms were written, people now use more abstract Arabic or Chinese scripts, and most Arabs cannot read the Chinese script, most Chinese cannot read the Arabic script, and most of the rest of the world can read neither. Moreover, even fluent readers still tend to understand, recognize, and recall better and faster what pictures depict than what the corresponding words in these readers’ own language mean (Kirkpatrick, 1894; Jenkins et al., 1967; Paivio and Csapo, 1973; Childers and Houston, 1984; Stenberg, 2006; Hockley, 2008; Quinlan et al., 2010; Curran and Doyle, 2011; Hockley and Bancroft, 2011; Carpenter and Olson, 2012; Rosenfeld et al., 2015; Baadte and Meinhardt-Injac, 2019; see also Cherry et al., 2008; Ally et al., 2009; Embree et al., 2012). This *picture superiority effect* suggests that, at least for concrete words that are easy to illustrate, an input language consisting of letters may be convenient enough for writers but an output language consisting of pictures is more helpful to readers.

The picture superiority effect does not always hold. When pictures are unclear, too detailed, or when photos or line drawings are used, which can be time consuming for the brain to process (Bühler, 2021), the very opposite effect can emerge (Snodgrass and McCullough, 1986; Amrhein et al., 2002; Whitehouse et al., 2006; Boldini et al., 2007; Defeyter et al., 2009; Hung et al., 2016). In fact, the images that are the easiest to digest are uncluttered, leave out irrelevant details, and only show the gist of a concept (Bühler, 2021)—that is, they are *icons* like those on road signs and on dashboards in cars and cockpits (McDougall et al., 2006; Lee et al., 2019; Shen et al., 2020; Friedrich et al., 2022).

The invention of hieroglyphs and Chinese characters depended on the creativity of small groups of scribes that operated locally with limited tools. By modern standards, quality was poor, and over time, writers were served better and better but readers worse and worse (Figure 1; Xiao and Treiman, 2012). Today, however, the design of the contemporary equivalents of pictograms and ideograms (emojis and more sleek, minimalist icons) engages talent from around the world, is facilitated by extremely flexible software, and is increasingly guided by ergonomic research focused on the reader rather than the writer (Bühler, 2021). People not only process such icons faster than alphabetic words but also find them more engaging (McDougall et al., 2016; Yangandul et al., 2018). Unsurprisingly, icons now abound in messages, manuals, advertisements, and on packaging, user interfaces, letterheads, and more (Katz et al., 2006; Koutsourelakis and Chorianopoulos, 2010; Andrews et al., 2011; Boelhouwer et al., 2013; Gawne and McCulloch, 2019; Sletvold et al., 2020; Liu et al., 2021; Prasetyo et al., 2021; Wu et al., 2022). What these icons depict is also not just concrete objects anymore; with great ingenuity, ever more abstract concepts are being visualized too.^3^

In a sign of the times, artist Bing (2013) recently managed to write an entire book using, instead of words, only icons. It was crafted with difficulty, no doubt. Yet even without learning any vocabulary, it can be read—or at least deciphered—by anyone, regardless of their linguistic background. Meanwhile, a number of scholars, scientists, artists, and ordinary citizens (Okrent, 2010) are using pictures as words in more systematic ways than does Bing. Some of them have artificially constructed pictorial “languages” (Nawar, 2020; Bühler, 2021), including *Pictoperanto* (Gros, 2011), *iConji* (Nawar, 2020)^4^, *Toki Pona* (Lang, 2014), *Minspeak* (Baker, 1982), *Picture Exchange Communication System* or *PECS* (Frost, 2002; see also Lancioni et al., 2007; Almurashi et al., 2022), *Blissymbolics* or *Semantography* (Bliss, 1978; Jennische and Zetterlund, 2015)^5^, *Ikon* (Meloni et al., 2022), *Nobel* (Randic, 2019), *aUI* (Weilgart, 1979), *IconText* (Beardon, 1995), and *Isotype* (Neurath, 2010; Burke, 2013).

Of these modern pictorial languages, the oldest is Isotype, which experienced its heyday in the first half of the 20th century. Its icons set the standard with their exceptionally ergonomic and artistic design. Most of the other iconic languages are inspiring in their own ways too. Still, as languages, they do have major shortcomings. First, some use icons that are almost abstract symbols, which makes them relatively hard to learn (Toki Pona, Blissymbolics, Nobel, aUI). They are easy to write by hand, but this activity is almost a thing of past. Second, some use overly colorful or cluttered icons (iConji, PECS, Minspeak, Ikon), which may appeal to children but makes these icons harder for the brain to process (Bühler, 2021). Third, most rely on a set of root words that must be combined to get additional words, but because this set is typically quite small, the combinations tend to become unwieldly (Okrent, 2010). Fourth, most are vague about how they can be both precise and concise (Okrent, 2010; Morin, 2022). On electronic devices, IconText does deal with this problem, and lets the reader go back and forth between different levels of detail in the same passage. Fifth, some have a rather limited scope, like mostly visualizing social and economic statistics (Isotype), exploring a Taoist worldview (Toki Pona), or communicating with people who cannot speak, write, or read (Blissymbolics, PECS, Minspeak). And sixth, none has a grammar that is well-defined or comprehensive enough for sophisticated use (Okrent, 2010; Morin, 2022).

### 1.3. The rebus principle accommodates writers, not readers

Early in the history of writing, before they started cutting corners with their drawing of hieroglyphs, ancient scribes made things easy on themselves by recycling old pictorial words to make new ones. As is still the case in modern Chinese, these new words often consisted in two simpler words, one with a relevant meaning but an irrelevant pronunciation (henceforth: *meaning component*) and one with a relevant pronunciation but an irrelevant meaning (henceforth: *pronunciation component*). Readers were expected to ignore these components’ irrelevant aspects. A study of Chinese readers, however, shows this is easier said than done (Yeh et al., 2017; see also O’Seaghdha and Marin, 1997; Zhou and Marslen-Wilson, 1999a,b; Yeh and Li, 2004; Chen and Yeh, 2017).

The study consisted of several “Stroop” experiments and presented colored Chinese characters (Yeh et al., 2017). As is typical in such experiments, participants were to name the color of these characters but ignore their meaning and pronunciation. Occasionally, however, a character was presented whose pronunciation component’s irrelevant meaning was associated with a color, and sometimes this color did not match the character’s color. One of these characters, for example, was “guess,” 猜, presented in yellow, which has a pronunciation component that means “cyan,” 青. Another was “pity,” 恤, also presented in yellow, which has a pronunciation component that means “blood,” 血. Blood is not a color; it is merely associated with one. Nonetheless, in both cases the irrelevant meaning of the pronunciation component put the participants on the wrong foot and slowed down their ability to name the color of the characters (here the color yellow).

The recycling of existing pictorial words to represent the sound, but not the meaning, of new pictorial words is known as the *rebus principle*. Ancient Sumerian, ancient Egyptian, and ancient Chinese all made use of it and modern Chinese still does. Across the world, however, words with the same meaning often sound different. Thus, languages that use the rebus principle are never inherently international. Moreover, because the rebus principle—as shown above—implants relevant but also irrelevant and potentially misleading associations in readers’ minds, languages that use the rebus principle are also never inherently reader-friendly. Unlike natural pictorial languages, therefore, neither Icono, nor any of the pictorial artificial languages mentioned earlier, make use of the rebus principle.

In contrast, the recycling of existing pictorial words to represent the meaning, rather than the sound, of new pictorial words can be useful. Chinese, for example, uses a square, 口, as a pictogram of a mouth to express the word “mouth” but recycles this square, in reduced size, in speech-related words like 詞, 句, and 信, which, respectively, mean “word,” “sentence,” and “letter.” Unfortunately, the Chinese words that have a pictogram in common often do not have any sounds in common. In Icono’s own bespoke pronunciation (see Supplementary material), icons are assigned a pronunciation, the pronunciation of words is dictated by that of their constituent icons, and words that share an icon also share the sound associated with it in their respective pronunciations.

### 1.4. Overview

Now, I lay out the foundation of *Icono*—a novel, icon-based language that remedies the downsides of both ancient and modern pictorial languages. The icons used for this endeavor have been obtained from artists from across the globe.^6^^,^^7^ I will show that, with such icons, Icono can in principle match the expressive power of Chinese—enough, that is, not just for informal messages but also for legal, literary, and academic communication. Section 2 lays out the ideas behind Icono’s vocabulary (Section 2.1) and sentence structure (Section 2.2); Section 3 describes two efficient ways to write Icono; Section 4 discusses the potential benefits of Icono for people with special needs, like those with dyslexia, aphasia, cerebral palsy, and autism with speech impairment; and Section 5 explains how Icono can become a success despite the fact that Esperanto and all its competitors have failed.

Icono is bound to make written communication particularly easy, both within and especially across borders, for mainly three reasons. First, like the languages of logic and mathematics, and unlike such languages as English or Esperanto (Zamenhof, 1909), Icono can be used for reading and writing even if one never learns its pronunciation or phonetic spelling (for those, see Supplementary material). Second, unlike such languages as English or Esperanto, but also unlike those of logic and mathematics, almost all of Icono’s words illustrate their own meaning. Some of these illustrations are self-evident, the meaning of others has to be learned, but none are arbitrary: they all function as visual mnemonics to aid recall (see also Chang et al., 2022; Weerasinghe et al., 2022). And third, unlike that of any other language, Icono’s sentence structure is revealed when it is most helpful: before, rather than after, one begins reading.

## 2. The nuts and bolts of Icono

### 2.1. Words that illustrate their meaning

#### 2.1.1. Combining concepts

**Figure 2:**
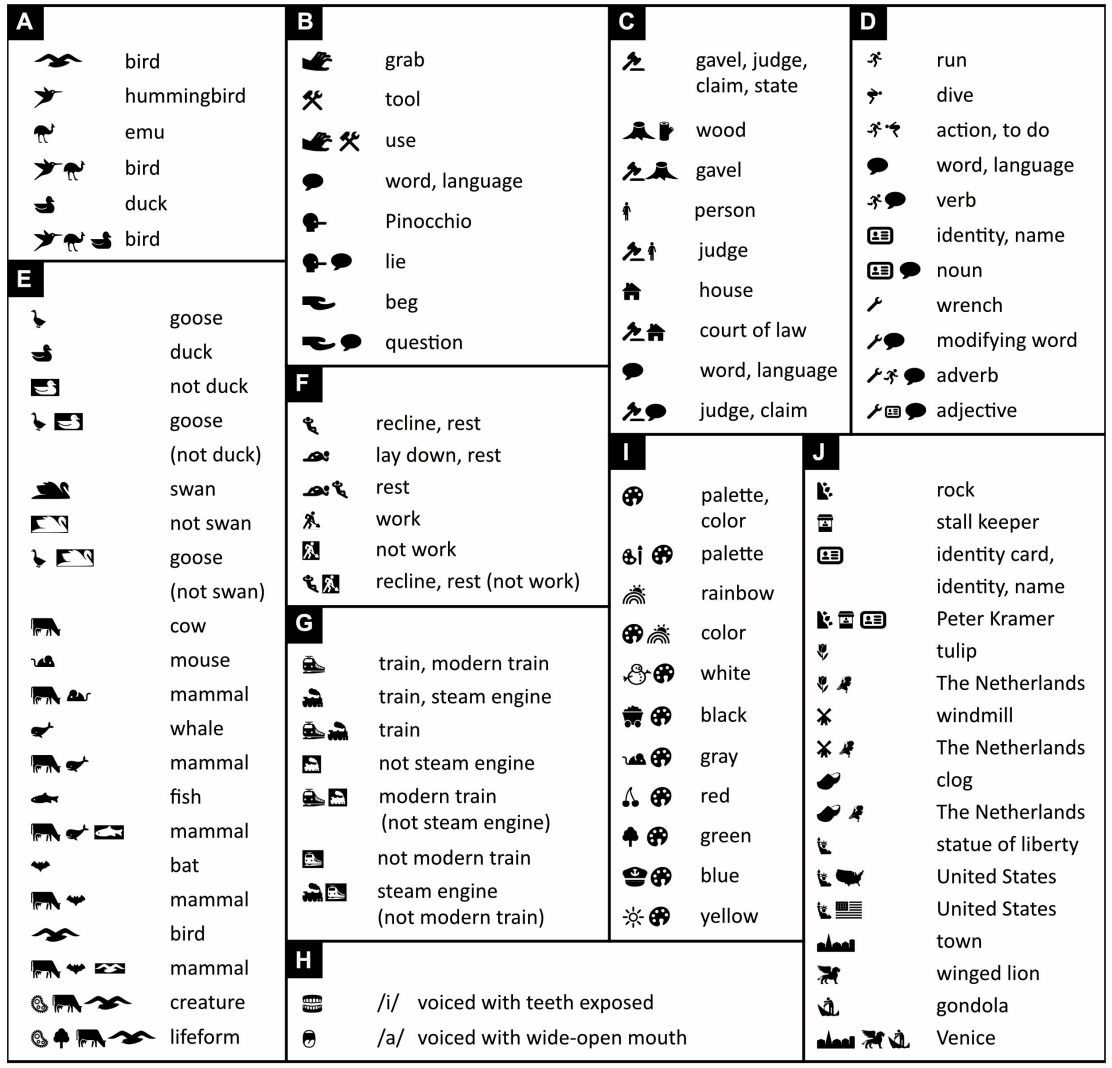
Word construction in Icono. Within each panel, iconic words are shown along with their English translations. Icons in white on black represent the negated versions of identical ones in black on white—a black duck on a white background means “duck,” a white duck on a black background “not duck.” Icono is rich in synonyms; note, for example, in panel E, the five different ways in which one can express the word “mammal.” For names that do not have any meaning, icons can be used that instead illustrate their own pronunciation, like those in panel H pronounced as “ee” and “ah,” (respectively, /i/ and /a/ in the International Phonetic Alphabet; for more, see Supplementary material). Note that “creature” is defined as any kind of microbe or animal, and “lifeform” as any kind of creature or plant. (Icons reproduced with permission from thenounproject.com; acknowledgments and links: https://osf.io/yrb49/).

The simplest words in Icono consist of a single icon with a prototypical illustration of its meaning (Rosch et al., 1976; see also Wittgenstein, 2009 but *cf.* Edwards et al., 2022). Writers, for example, can opt to write the word “bird” with a single icon of an average-looking bird (Figure 2A).

Still, whereas words that consist of a single icon are just fine in some contexts, in others they may be ambiguous or suggest a narrower meaning than intended (see also Lupyan and Winter, 2018). To solve the problem, just like Chinese characters combine pictograms and ideograms (radicals), Icono combines icons. A synonym for “bird,” for example, is obtained by putting together an icon of a hummingbird and an icon of an emu—their combination’s meaning given by what they have in common. This icon combination does not reveal its meaning quite as readily as does a prototype-icon but is still easy to learn and tells us that a generic bird is meant, not any specific species. Various natural languages, including Sanskrit and Greek, also combine pairs of relatively concrete words (here “hummingbird” and “emu”) to obtain more abstract ones (here “bird”). (Such pairs are called *dvandva compounds*, after the Sanskrit word for “pair”: *dvandva*, द्ंद्.)

In Icono, the core of a word is predefined and fixed. Yet, like in Chinese to some extent, but unlike in most other languages, writers can always prolong and often shorten a word to make it, respectively, less ambiguous or more concise. So, for extra clarity, writers can choose to add an extra icon of, say, a duck or a swan to those of a hummingbird and an emu to further drive home “bird” is meant (Figure 2A). Once the reader can be assumed to have gotten the message, the extra icon can then be dropped again.

The icons that make up a word in Icono need not be independent instances of the concept this word expresses. They can also complement each other. For example, the combination of the icons of a grabbing hand and a set of tools expresses the word “use” (Figure 2B). Alternatively, borrowing from Sumerian and ancient Egyptian, one can use a “determinative”—a representation of a word’s broader semantic category (Fischer, 2001; Coulmas, 2003; Nawar, 2020). For example, to express the meaning of words having to do with judgments, Icono uses an icon of a gavel (a judge’s wooden hammer). To this gavel icon, Icono then adds an icon that depicts wood for “gavel,” person for “judge,” house for “court of law,” speech blurb for “judgment, claim, or statement,” and so on (Figure 2C). A speech blurb indicates relevance to language and can as such be used in other words too. For example, in Chinese, nouns are called “name words,” 名詞, and in Icono, considering that names are used as identity labels, “noun” is expressed with the icons of an identity card plus a speech blurb (Figure 2D). Verbs, in Chinese, are called “action words,” 動詞, and in Icono, “verb” is expressed with the icons of an active person (a runner) plus, again, a speech blurb (Figure 2D).

#### 2.1.2. Comparing and contrasting concepts

Unlike any other language, Icono not only uses positive hints to word meaning, expressed with black icons on a white background, but also negative ones, expressed with white icons on a black background. If more clarity is needed to express the concept of a goose, for example, one can add to its black-on-white icon an additional white-on-black one of a duck or a swan (Figure 2E). This would signal that a goose is meant, not a similar-looking duck or swan. Likewise, a word like “mammal” can be expressed with two black-on-white icons of two radically different instances of this concept, like a cow and a mouse. Yet, one can also choose a synonym consisting of the black-on-white icons of a cow and a whale, or a cow and a bat, and then—for extra clarity—temporarily add a white-on-black icon of a fish or a bird (Figure 2E).

The Chinese word for “mammal,” 哺乳動物, is its own definition and means “breast-feeding animal.” The definition is clear, but the word is two characters longer than most Chinese words and contains 11 pictograms and ideograms (radicals). At a normal reading speed, this is rather a lot for readers to digest. Especially the earliest designers of artificial languages also often used words that defined themselves in terms of root words. The result proved unwieldly (Okrent, 2010). A word in Icono therefore typically consists of icons that offer key mnemonics to this word’s meaning but not a full-fledged definition. This way Icono can express “mammal” with just two icons, or temporarily with one or two additional ones, rather than with almost a dozen.

#### 2.1.3. Keeping words short and simple

An important question is whether Icono’s vocabulary could ever rival that of a full-fledged, natural language like Chinese, and how many icons per word would be needed in this case. Most Chinese words consist of two characters, and each character usually consists of substantially fewer than 10 radicals. Chinese words thus typically consist of at most 2 × 10 = 20 pictograms and ideograms. Chinese restricts its choice of radicals to only some 200. Suppose, for argument’s sake, that Icono replaced each radical with a modern icon and used no other icons. In this case, to rival the expressive power of Chinese, it could need up to 20 icons per word. Unlike Chinese, of each icon, Icono uses both a white-on-black (positive) version and a black-on-white (negative) one. Taking this into account, Icono could need up to 10 icons per word rather than 20. Importantly, however, Icono allows the use of many more than just 200 icons. Hence, if it adopted, say, 1,000 different ones, it could in principle get by with on average only 10/(1000/200) = 2 icons per word. Indeed, in Chinese, the word for “rest,” 休, is expressed with two radicals, which, respectively, represent a person, 人, and a tree, 木 (think of person leaning against a tree). In Icono, instead, a single icon of a resting person can suffice on its own (Figure 2F). Of course, if more clarity is needed, an additional icon can always be added, like a positive icon that expresses rest in a different way or a negative icon of work (Figure 2F).

Typically, the more abstract a concept, the more icons are needed to express it unambiguously. “Mammal,” for example, can be expressed with the combination of the icons of a cow and a mouse, but “cow” and “mouse” themselves each require only one icon (Figure 2E). Still, once the reader has been presented with a long version of an iconic word, and can be assumed to have understood it, this word can subsequently often be abbreviated without much loss in clarity. The Chinese word for “train,” 火車, for example, translates to “fire vehicle” (think of a coal-fired steam engine). Yet once the reader can be assumed to have understood that a train is meant, and not a car or a bus, the Chinese often abbreviate “fire vehicle” to just “vehicle.” Context may also help: when someone looks at a departing train and says “vehicle is leaving,” it is clear that a fire vehicle is meant, no need to spell this out. Of course, Icono is not limited to some 200 radicals and can express the concept of a train more concisely, not with a fire-pictogram plus a vehicle-pictogram, but simply with a single icon of a train (Figure 2G). Still, if one wishes, one can initially use the black-on-white icon of a modern train and a white-on-black one of a steam engine (Figure 2G) to signal that a modern train is meant rather than just any kind of train. Subsequently, the icon of the modern train by itself will do.

#### 2.1.4. Dealing with meaningless names

In Icono, words typically illustrate their own meaning. Yet one should be able to express proper names too, and these may not necessarily have any meaning. Borrowing an idea from Korean (see Supplementary material), one solution is to use icons that illustrate their own pronunciation—that is, icons that refer to sounds and at the same time also illustrate how to use the mouth to produce these sounds (Figure 2H; Supplementary Figure S1). The problem is that names that for some are easy to pronounce, for others are often a major tongue-twister. English speakers, for example, tend to ignore the all-important intonation of Chinese names and Chinese speakers struggle with the consonant clusters and unusual vowels of English. A user-friendly international language, therefore, does not preserve the original pronunciations of names but adjusts them so that they become less susceptible to mispronunciation and miscommunication.

Given such problems, however, one could consider coining meaningful alternatives to meaningless, or seemingly meaningless, names. For most Chinese speakers, for example, my name “Peter Kramer” is very hard to pronounce. Yet “Peter” derives from “rock” and “Kramer” from “stall keeper,” and neither is difficult to depict with icons that illustrate themselves and are easy to use worldwide (Figure 2J; for an etymology of names, see: www.behindthename.com). My native The Netherlands is well known for its tulips, windmills, and clogs. An alternative name for this country could thus be an icon of the geographical contours of The Netherlands plus an icon of a tulip, a windmill, or a clog (Figure 2J). Likewise, “United States of America” can be expressed with an icon of the statue of liberty plus an icon of the US’s contours or distinctive flag, and “Venice” with the icons of a town, a winged lion, and a gondola (Figure 2J). Elderly people in particular have trouble recalling meaningless proper names (Evrard, 2002). This, then, may be another good reason to replace such meaningless names with meaningful ones.

To ensure that color names are printable in black and white and readable by the colorblind, Icono expresses them with the icon of a painter’s palette plus an icon of something associated with these colors, like a snowman for “white,” cherries for “red,” a tree for “green,” and a cart full of coal for “black” (Figure 2I).

#### 2.1.5. Dealing with abstract concepts

**Figure 3:**
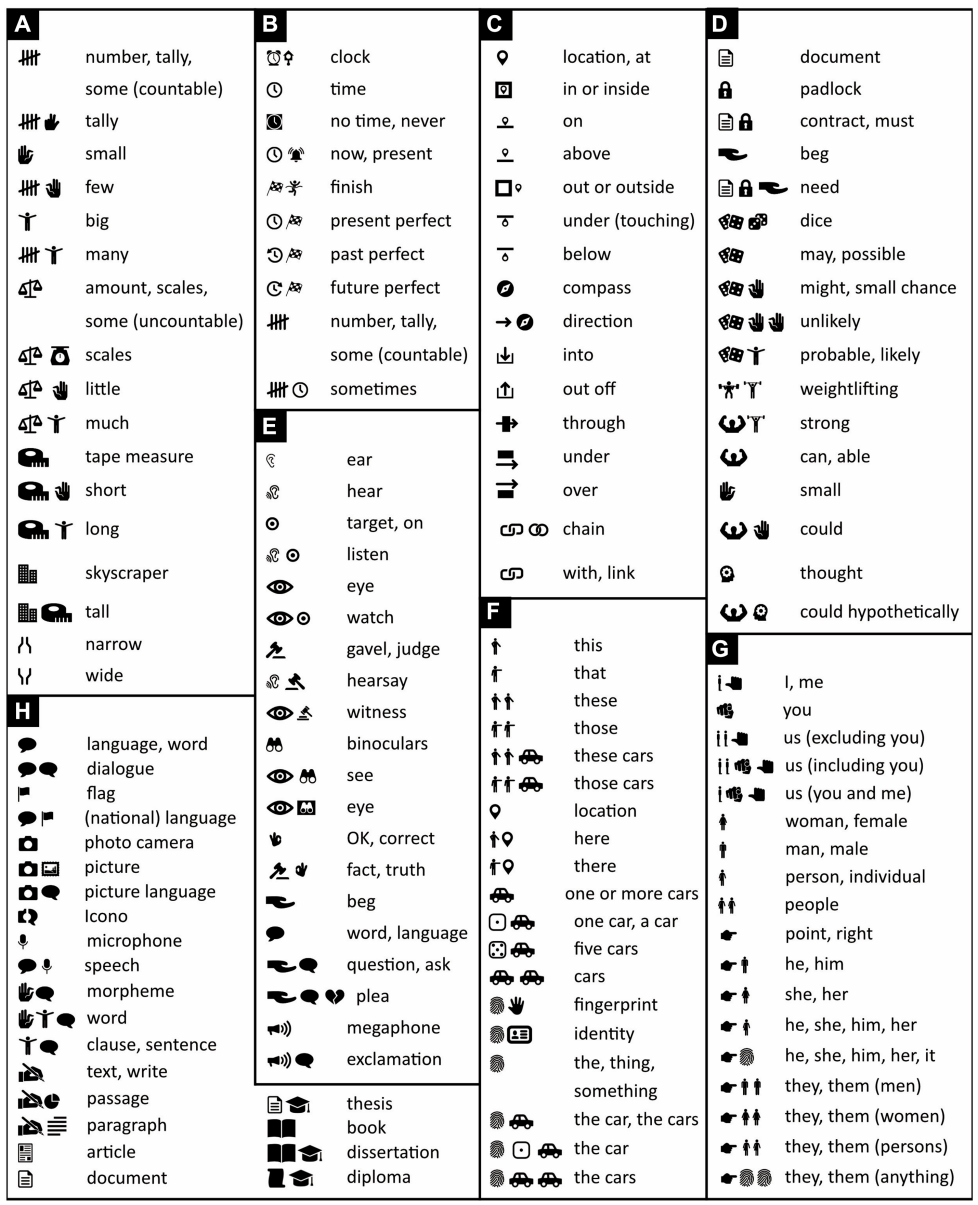
Frequently used abstract words. Iconic words and their translations regarding **(A)** quantity; **(B)** past, present, future (tense), and completion (aspect); **(C)** prepositions; **(D)** possibility and probability; **(E)** whether something is hearsay, a question, a plea, an exclamation, etc. (mood); **(F,G)** pronouns like “he,” “she,” “this,” “that” and determiners like “this” and “that” but also “the,” “one,” “five” (nouns accompanied by determiners are placed between brackets and multiple determiners are separated from one another with commas, but more about this later); and **(H)** language-related concepts. An icon of a hand pointing to the right **(G)** means “to point” if used as a verb or as part of a pronoun, otherwise it means “right.” (Icons reproduced with permission from thenounproject.com; acknowledgments and links: https://osf.io/yrb49/).

The icons whose meanings are easiest to learn from scratch are those that are tightly related to the concrete everyday impressions and experiences we have had since early childhood (Bühler, 2021; see also Shen et al., 2020; Friedrich et al., 2022). Most of the icons in Figure 2 are of this type. The more abstract the concepts that icons represent, the less often their representation approaches this ideal (but see Scicluna and Strapparava, 2019). Some tokens are pictorial but nonetheless abstract. The “heart” icon, for example, is widely understood to mean “love” but neither depicts any emotion nor much resembles any physical heart. Because its meaning is thus little more than a convention, it is strictly speaking a symbol rather than an icon (Shen et al., 2020; Bühler, 2021). Similarly, a speech blurb is a symbol but is widely understood to stand for words or language (Figure 3H), and the location indicator (Figure 3C) and OK gesture (Figure 3E) have become quite international symbols as well. Following Bing (2013), until more intuitive alternatives become available, Icono also uses such well known, icon-like, but nonetheless quite abstract pictures (Figure 3).

Remarkably, although formal logic and mathematics are often deemed quintessentially abstract, versions of them already exist that are to a large extent iconic rather than symbolic (for *iconic logic*, see Roberts, 1973; Kauffman, 2001; Shin, 2002; Dau, 2011; Peirce, 2020; Peirce, 2021a,b; Bricken, 2023; Kramer, 2023; for *iconic mathematics*, see Spencer Brown, 1969; Kauffman, 1995; Bricken, 2019a,b; Bricken, 2021; Kramer, 2022).

It has, in fact, been argued that even the most abstract concepts, including mathematical ones, are *embodied*—directly or indirectly grounded (Barsalou et al., 2018; Borghi, 2022) in concrete physical experiences or action preparations (Lakoff and Núñez, 2000; Gallese and Lakoff, 2005; see also Fischer et al., 2021; Glenberg, 2021). Still, the more abstract a concept, the more diverse the items or events across which this concept generalizes (Del Maschio et al., 2021) and the harder it is to visualize this concept with just a few icons (see also Lupyan and Winter, 2018). For example, whereas the concept of a cow generalizes over all cows, the concepts of mammal, creature, and lifeform generalize not only over all cows but also over progressively larger numbers of other species. Hence, whereas it expresses “cow” with just a single icon, Icono uses two icons to express “mammal,” three to express “creature,” and four to express “lifeform” (Figure 2E). Likewise, whereas the concept of running generalizes over many physical actions, the concept of action generalizes over many more and requires an extra icon (Figure 2D). Particularly hard to illustrate succinctly are concepts like “and,” “or,” “because,” “therefore,” which apply to a great many extremely different situations. Exactly for these kinds of concepts, mathematics and logic reserve abstract symbols. Because these are already widely used all around the world, Icono lets pragmatism triumph over dogmatism, and adopts some of these symbols too (see next section).

### 2.2. Sentences that reveal their structure

#### 2.2.1. Structuring sentences graphically

For Icono to become a suitable world language, not only its words but also its grammar must be easy to learn. For the most part, Chinese uses vocabulary rather than grammar to distinguish plural from singular, and present from future and past; and it uses neither grammatical case (e.g., no distinction between “they” and “them”) nor grammatical gender (e.g., no distinction between linguistically “male” or “female” words). Icono would thus be relatively easy to learn if it adopted Chinese grammar. The grammars of Austronesian languages like Malay and Hawaiian would be excellent alternatives, and the grammars of some non-pictorial, artificial languages like Esperanto are not too bad either.

For two reasons, however, Icono has its own grammar. First, languages often use conjugation, particles, or affixes to express their grammar. Yet, as a kind of training wheels, language-instruction material often uses graphical tools like highlighting, italics, and boldface to distinguish grammatically distinct passages before they are read. As such training wheels are quite helpful, Icono turns them into a permanent feature of its script and forgoes the use of most linguistic markers of grammar. Thus, unlike any other language, Icono reveals the structure of its sentences graphically before, rather than linguistically after, one begins reading. As a side effect, Icono need not constrain word order much and writers can adjust this order either to what they are accustomed to in their native language, to what they deem most important, or to what burdens readers the least (Hahn and Xu, 2022). Second, although the grammars of languages like Chinese, Malay, and Hawaiian are relatively simple, without sacrificing functionality, they could be simpler. The same is true for the Indo-European-style grammars of artificial languages like Esperanto.

#### 2.2.2. Separating, organizing, and connecting words

**Figure 4:**
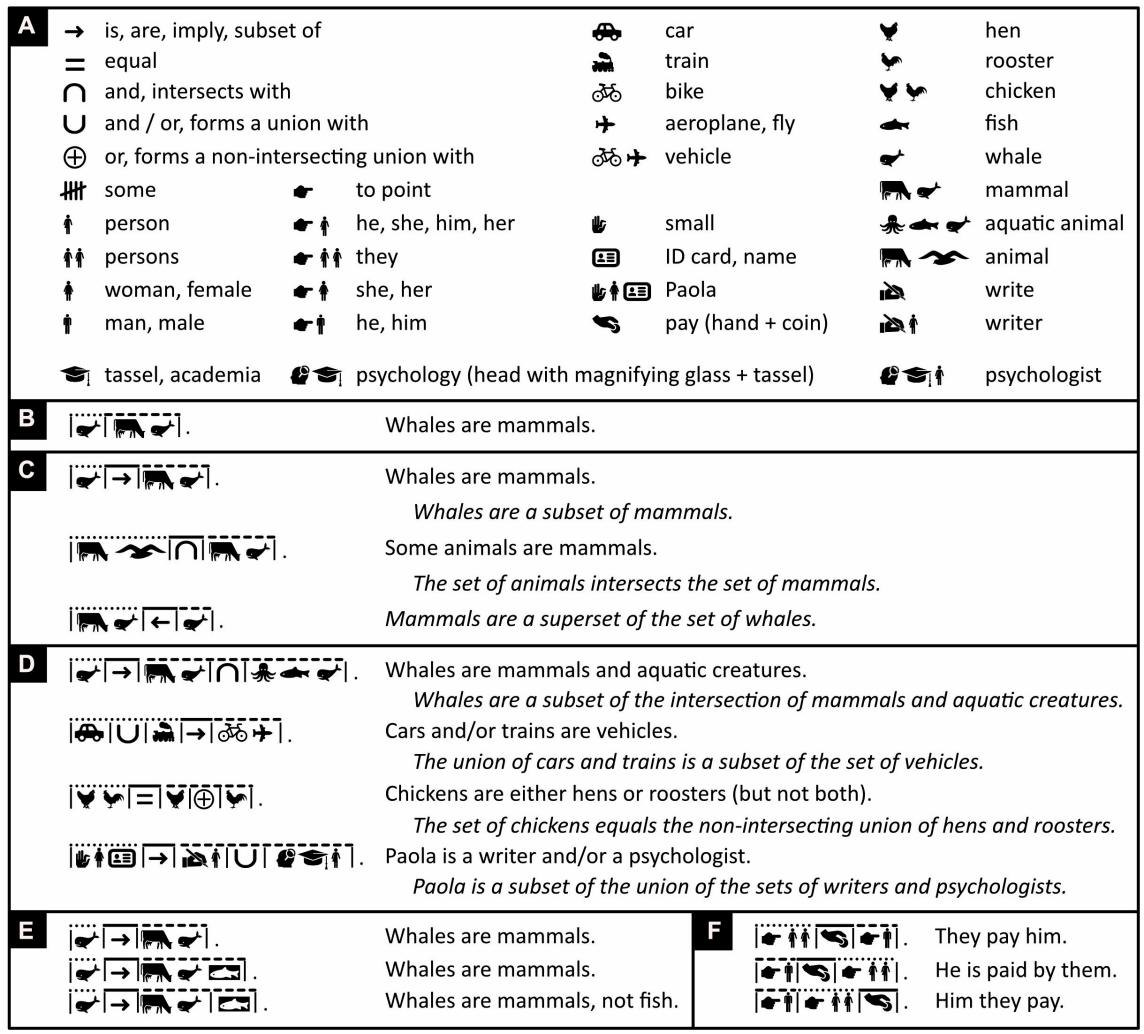
Separating and connecting words. **(A)** Mathematical and iconic vocabulary used in the panels below, plus some associated words. Because whales are discussed in panels **(B–E)**, the word “mammal” is expressed with the icons of a cow and a whale rather than a cow and a mouse (Figure 2E), but either alternative is fine to use. **(B–F)** By default, with the exception of determiners like “a,” “the,” “some,” words are separated from each other by vertical bars. Each sentence’s subject, verb, and object or subject complement is placed underneath, respectively, a dotted, a continuous, and a dashed line. **(B)** Where possible, the verb “to be” is omitted. **(C–E)** Where it is not omitted, “to be” is translated with logical or mathematical symbols. **(D)** Icono’s version of “Paola” reflects the fact that this name derives from Latin for “small.” **(C–D)** Loose English translations are given in regular font, literal ones in italics. **(E)** Words can be clarified by adding negative (white-on-black) icons to them. The first two lines are thus synonymous; the first is more concise, the second less ambiguous. Abutting icons form a single word; those separated by a vertical bar or punctuation mark belong to different words (compare lines 2 and 3). **(F)** Word order in Icono is relatively free. There is no need for passive sentences and no need to use words like “by” to express them. (Icons reproduced with permission from thenounproject.com; acknowledgments and links: https://osf.io/yrb49/).

Like English words, Chinese characters are separated from each other by a little bit of space. This was good enough in ancient times, when one character was one word (Fischer, 2001). Now, however, Chinese words often consist of two characters and sometimes more, and there is nothing that separates them from each other. Modern Chinese words must thus be recognized before they can be distinguished. Icono’s words, instead, are visibly separated from each other by either a vertical bar (Figures 4B–F) or a punctuation mark (shown later) and can thus be distinguished before they are recognized.

Sentences typically have up to three major parts that, in most languages, are known as subject (e.g., “*he* loves her”), verb (e.g., “he *loves* her”), and either object (e.g., “he loves *her*”) or subject complement (e.g., “he is *a man*” or “he is *tall*”)—the subject complement, unlike the object, offers information about the subject. To ease parsing, Icono divides its sentences into these three parts too and places above them, respectively, a dotted, a continuous, and a dashed line (Figure 4).

**Figure 5:**
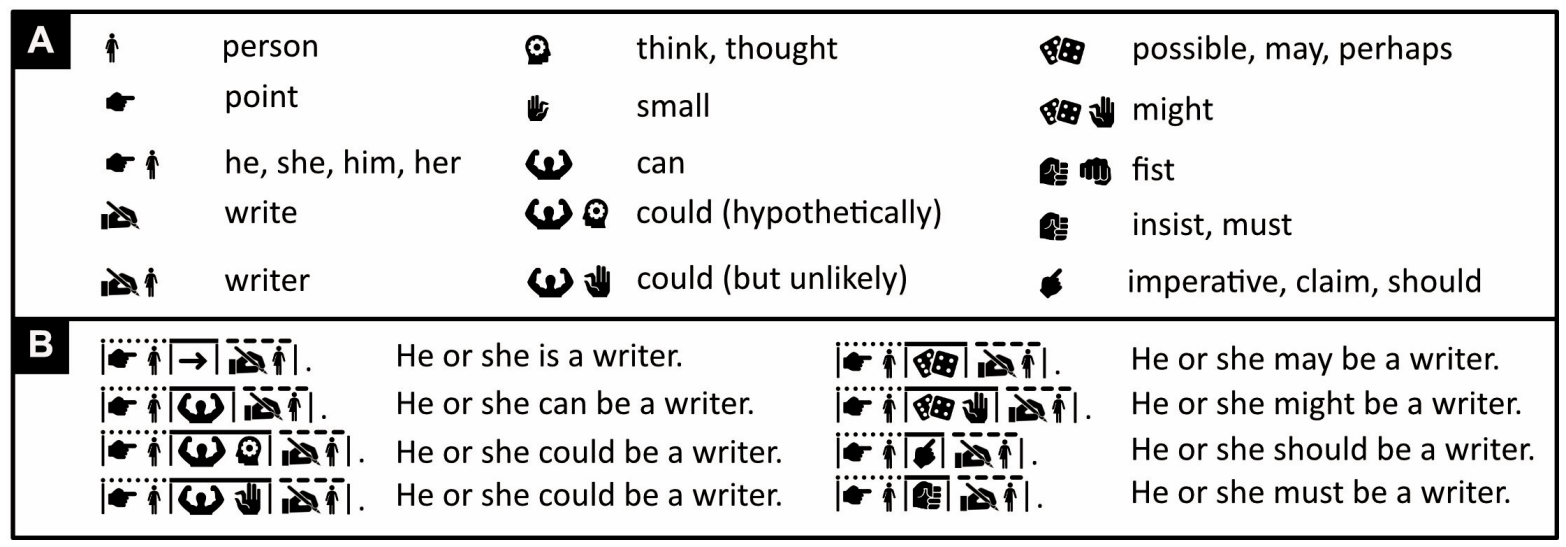
Expressing uncertainty. **(A)** Iconic vocabulary used in the panel below, plus some associated words. **(B)** The first sentence contains the verb “to be,” the following ones so-called “modal” alternatives that express different degrees of uncertainty, like can, could, may, might, should, and must. (Icons reproduced with permission from thenounproject.com; acknowledgments and links: https://osf.io/yrb49/).

Verbs typically connect subjects to objects or complements. Especially in Austronesian languages like Malay, however, the verb “to be” is often omitted, and “he is big” and “he is a man,” for example, simply become “he big” and “he man.” Icono embraces such economy of words (Figure 4B). Still, whether stated explicitly or implicitly, “to be” is ambiguous; it can mean “to be equal to” but also “to be a subset of.” “Roosters are male chickens,” for example, is interpreted to mean “roosters equal male chickens” but “whales are mammals” is interpreted to mean “whales are a subset of mammals.” Hence, where more clarity is needed, Icono translates “to be” with the help of logical or mathematical symbols representing logical or set relationships like “imply,” “is a subset of,” “intersects” and so on (Figures 4C–E). In Icono, these logical and set relationships are interchangeable; “being a whale implies being a mammal,” for example, means the same thing in Icono as “whales are a subset of mammals” (Figure 4C). As alternatives to the verb “to be,” Icono offers iconic versions of so called “modal” words like “may,” “might,” “could,” “must” (Figure 5).

In English, the connectives “and” and “or” are ambiguous too. For example, “Whales are mammals *and* aquatic animals” amounts to saying, “whales are mammals that are aquatic animals,” but “cars *and* trains are vehicles” is not equivalent to saying, “cars that are trains are vehicles.” Similarly, with “chickens are hens *or* roosters” we may mean “chickens are hens or roosters but not both,” yet we usually do not consider “Paola is a writer or a psychologist” false if Paola is both a writer and a psychologist. Icono also uses set notation to express and distinguish the former *exclusive-or* and the latter *inclusive-or* (Figure 4D). For attempts to exorcize all syntactic and all semantic ambiguity, see, respectively, the non-pictorial artificial languages of *Lojban* (Sampson, 1999; Cowan, 2015; www.lojban.org) and *Ithkuil* (Quijada, 2011). For a discussion of why, however, such extremism becomes unwieldly, see Okrent (2010) and Garvía and Soto (2015).

#### 2.2.3. Modifying words

**Figure 6:**
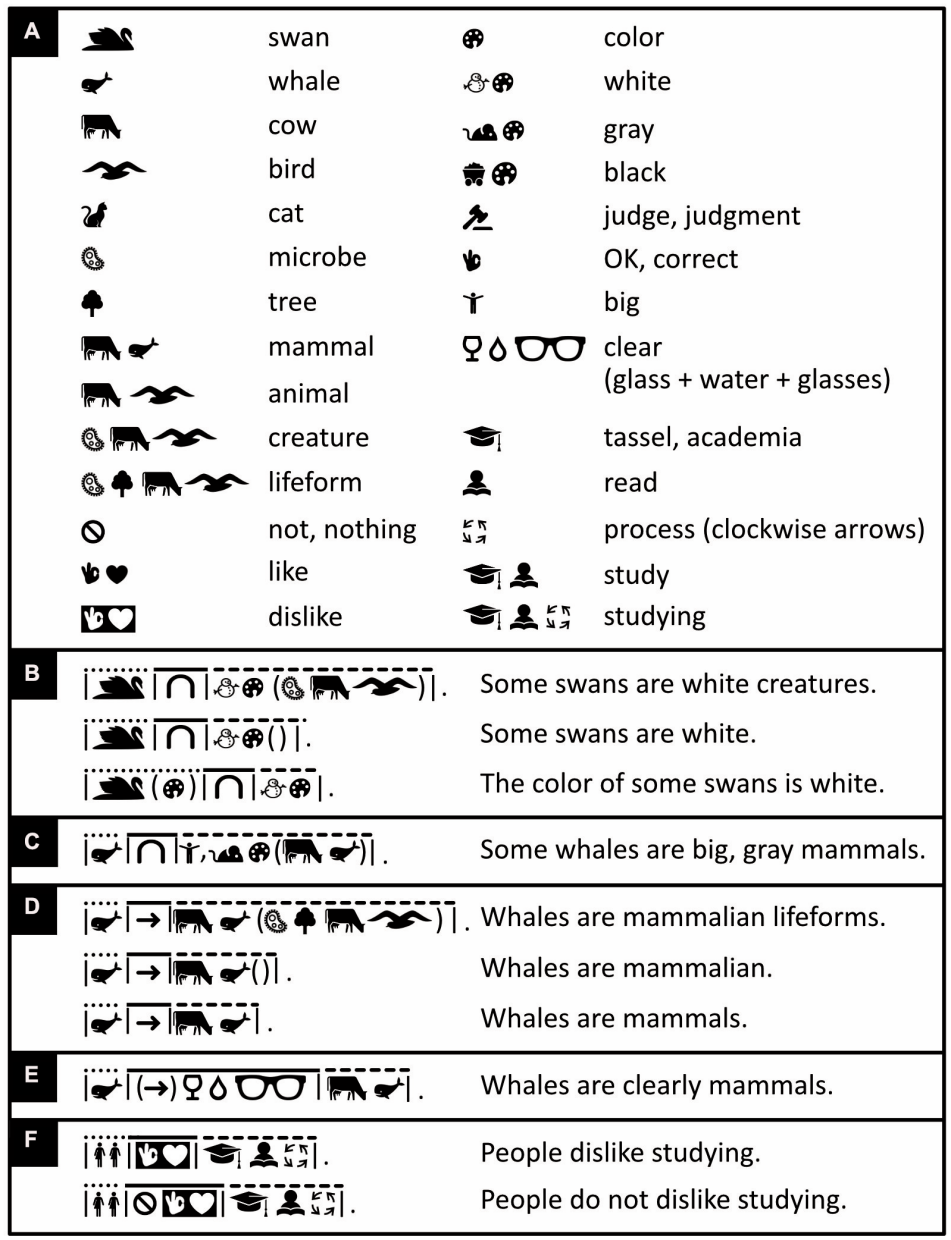
Modifying words. **(A)** Iconic vocabulary used in the panels below, plus some associated words. **(B)** Modified words (nouns, verbs) are placed between brackets; modifying ones (adjectives, adverbs) precede or follow these bracketed words. **(C)** Different modifiers of the same noun or verb are separated from one another by commas. **(B,D)** Modifiers of omitted nouns or verbs either precede or follow brackets enclosing an empty space. **(E)** An example of an adverb (here “clearly”). **(F)** An example of negation and another of double negation. (Icons reproduced with permission from thenounproject.com; acknowledgments and links: https://osf.io/yrb49/).

Nouns and verbs can be modified by adjectives and adverbs. In Icono, to graphically distinguish the modifier from the modified, the modified is placed between brackets and the modifier either precedes or follows it, outside these brackets (Figure 6). The brackets are used in a way similar to those in mathematical functions like *f*(*x*): nouns and verbs take the place of the variable *x*, adjectives and adverbs of the function *f*, and the resulting modified nouns and verbs of *f*(*x*) as a whole.

Modifiers can sometimes modify words that, for the sake of brevity, are not explicitly stated. For example, “some swans are white” is short for something like “some swans are white creatures.” In Icono, to ensure modifiers of unstated words can be recognized as modifiers, and are not mistaken for nouns, they either precede or follow an empty pair of brackets. Thus, “white” in Figure 6B needs to be accompanied by brackets in line 2 but not line 3, and Icono distinguishes, say, “whales are mammalian” from “whales are mammals” (Figure 6D).

The word “not” can modify words too. In this case, however, the fore-and background colors of the modified words are reversed from black on white to white on black (Figure 6F). To express double negation, like in “People do not dislike studying,” the verb “like” is expressed in white on black and also preceded by the “forbidden” road sign (Figure 6F)—an alternative way of expressing “not.”

#### 2.2.4. Indicating time, completion, and intent

**Figure 7:**
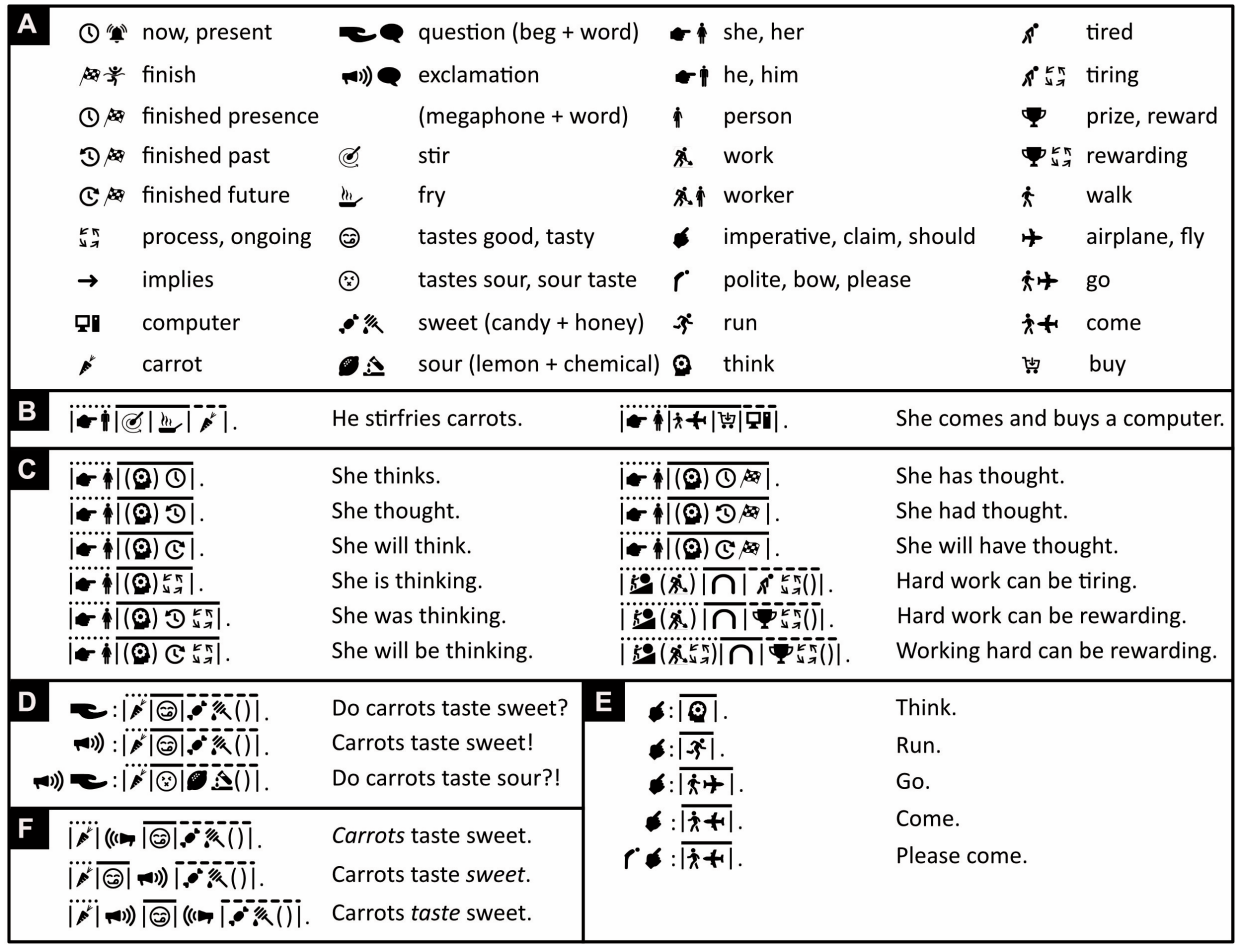
Indicating time, completion, and intent. **(A)** Iconic vocabulary used in the panels below, plus some associated words. **(B)** Verb serialization in Icono but not English (left and right) and verb contraction in English but not Icono (left). **(C)** Icono’s equivalents of grammatical tense (present, past, future) and aspect (finished, ongoing). **(D)** Icono’s equivalents of questions and exclamations. **(E)** Icono’s equivalents of other kinds of grammatical mood. **(F)** Icono’s expression of emphasis. (Icons reproduced with permission from thenounproject.com; acknowledgments and links: https://osf.io/yrb49/).

To express that events happen simultaneously or in close succession, English sometimes uses verb contractions like “stirfry,” “kickstart,” and “forcefeed.” Like many Bantu, Austronesian, and Papuan languages, Icono uses “verb serialization” instead, like in “He *stirs fries* carrots” (Figure 7B, left) and in “She *comes buys* a computer” (Figure 7B, right).

To varying degrees, many languages use grammar to express whether events occurred in the past, take place now, or will happen in the future (*tense*), and many more whether events have finished or are ongoing (*aspect*). Icono enables the expression of both tense and aspect (Figure 7C), but unlike English, mandates neither. In Icono, for example, like in Malay, “I work yesterday” is grammatical and unambiguously refers to the past. Similarly, although “whales are mammals” is a timeless truth, in English one must express it with the present tense. In Icono, instead, one uses grammatical features like tense only when they serve a purpose.

Sentences can be uttered for various reasons and be statements, questions, commands, suggestions and other kinds of so-called *mood*. In English and many other languages, questions and exclamations end with a dedicated punctuation mark. In Arabic, in contrast, questions often begin with a particle that informs readers upfront, rather than retroactively, that they are questions. Icono follows this example for not only questions but also all other expressions of mood (Figures 7D,E). Icono’s exclamation icon doubles as a kind of italics and can also be used to emphasize words or passages (Figure 7F).

#### 2.2.5. Expressing spatial and temporal relations

**Figure 8:**
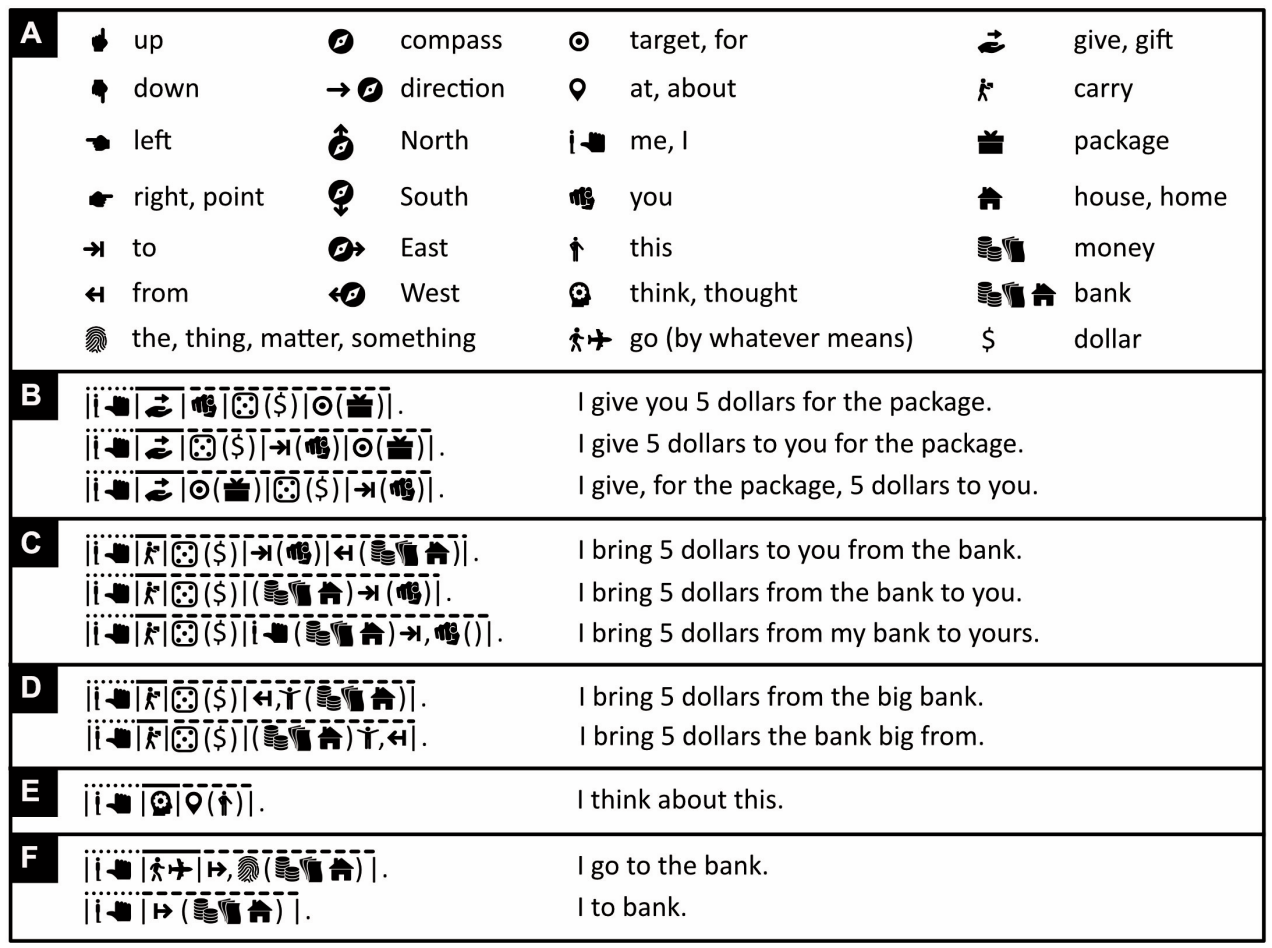
Expressing spatial and temporal relations. **(A)** Iconic vocabulary used in the panels below, plus some associated words. An icon of a hand pointing to the right means “to point” if used as a verb or in a pronoun, otherwise it means “right.” **(B)** Adpositions operate on expressions between brackets. Indirect adpositions must precede direct ones (line 1), otherwise word order is relatively free (lines 2–3). **(C)** Sometimes an adposition can operate on two nouns simultaneously (compare line 1 to lines 2 and 3). **(C,D)** Adpositions and adjectives operating on the same expression are separated from one another by commas. **(E)** The location icon is used as an adposition with the deliberately imprecise meaning of “somewhere at, around, in, on, under, above, near, with, about, or through something or some location.” **(F)** In Icono, both sentences are grammatical. (Icons reproduced with permission from thenounproject.com; acknowledgments and links: https://osf.io/yrb49/).

**Figure 9:**
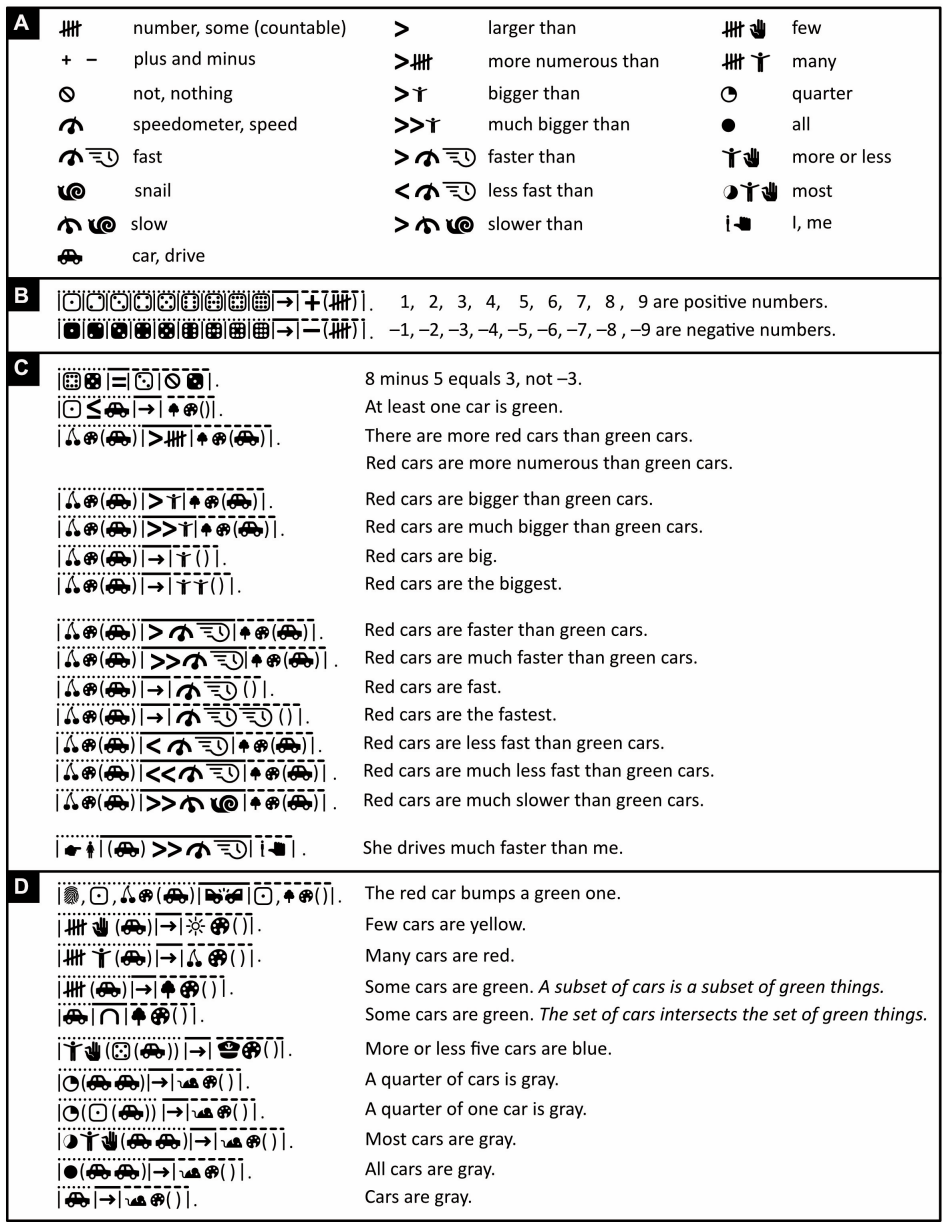
Dealing with quantity and identity. **(A)** Iconic vocabulary used in the panels below, plus some associated words. **(B)** Iconic versions of the numbers 1 through 9, along with their negative equivalents, used in two related examples (see Kramer, 2022). **(C)** Comparing quantities with the help of mathematical symbols and expressing degrees of quantities and superlatives with icon duplications. **(D)** Expressing the singular with the icon for the number 1 (a die with one dot) and the plural with icon duplication (e.g., two car icons rather than one). Expressing precise as well as vague quantities and using mini pie-charts to visualize proportions. Note that “some cars are green” translates to either “a subset of cars is a subset of green things” (i.e., being a member of this subset of cars *implies* being a member of the set of green things) or “the set of cars intersects the set of green things.” (Icons reproduced with permission from thenounproject.com; acknowledgments and links: https://osf.io/yrb49/).

Spatial and temporal relations are often expressed with *adpositions* (prepositions, postpositions) like in the English “*on* a ship,” “*in* a house,” “*through* the air,” “*before* sunrise,” “*after* nightfall,” and so on. In Icono, like in English, all grammatical objects, except the *direct* and *indirect* ones, are accompanied by an adposition. In “I bring you five dollars for the package,” the direct and indirect objects are, respectively, “five dollars” and “you.” To ensure the reader can tell which is which, in Icono, like in English, Chinese, and many other languages, the indirect object must always precede the direct one (Figure 8B). Like adjectives and adverbs (Figures 6, 7), in Icono, adpositions either precede or follow an expression between brackets (Figures 8B–E). The same is true for determiners like “a,” “the,” “one,” “two,” “three” (Figures 8F, 9D). Adpositions, adjectives, and determiners that operate on the same expression are separated from one another by commas (Figures 8C,D).

In abstract expressions in English like “*on* this matter,” “*in* matters like these,” or “*about* this issue,” the correct choice of adposition can seem rather arbitrary. To make things easier, whenever an adposition’s precise meaning is unimportant, Icono uses the location icon and assigns it the deliberately imprecise meaning of “somewhere at, around, in, on, under, above, near, with, about, or through something or some location” (Figure 8E). In the same spirit, more than does English, Icono omits needless words. For example, in Icono, “I go to the bank” is grammatical (Figure 8F, line 1), but one can get the main point across more succinctly with a Malay inspired “I to bank,” using neither verb nor definite article (Figure 8F, line 2). In Figure 8E, the verb is clearly essential, in Figures 8B–D it could probably also be eliminated.

#### 2.2.6. Dealing with quantity and identity

By themselves, the letters that make up words are just arbitrary squiggles, and the same is true for the digits that make up numbers. In Icono, instead, digits are represented with dots in dice-like configurations (Figure 9B), with the number of dots indicating numerical value (cardinality). Positive numbers are represented in black-on-white, negative ones in white-on-black (for more on iconic numbers and iconic mathematics, see Kramer, 2022). To be able to say something like “eight minus five equals three, not minus three,” the word “not” is expressed with the “forbidden” road sign (Figure 9C, line 1; compare with Figure 6F, line 2). Other relationships between numbers and quantities (Figure 9C) are expressed with the help of mathematical symbols like “=” (equals), “<” (smaller than), and “>” (larger than).

The number 1, expressed with a die containing one dot (Figure 9B), is used not only as a number but also as an *indefinite* article, meaning “a.” (Figure 9D, line 1). As a *definite* article, which refers to an already identified person or thing, Icono offers an icon of something associated with identity: the fingerprint (Figure 9D, line 1; see also Figure 3). By default, however, like in Russian, Chinese, Malay, and many other languages, the definite article is not used.

In Icono, unlike in English, but just like in Malay and mostly also Chinese, distinguishing singular and plural is optional rather than mandatory. In English, “sheep” refers to one or more sheep, but “car” to just one car and “cars” to more than one. In Icono, a single icon of a sheep or a car means “one or more sheep” or “one or more cars.” If desired, the singular form of a noun can be expressed by placing the iconic version of the number 1 before or after this noun, and the plural form—inspired by Malay—by duplicating any of the noun’s constituent icons (compare Figure 9D, lines 1 and 7). Duplication of any of the icons of an adjective or verb, rather than of a noun, expresses a superlative, like “biggest” or “fastest,” or a greater degree, like “*much* more” or “*much* less” (Figure 9C). Proportions like “a quarter” are expressed with small pie charts and vague quantities like “some” or “approximately five” with the icons of an indistinct tally or the gestures for big and small, which together mean “more or less” (Figure 9D).

#### 2.2.7. Nesting sentences

**Figure 10:**
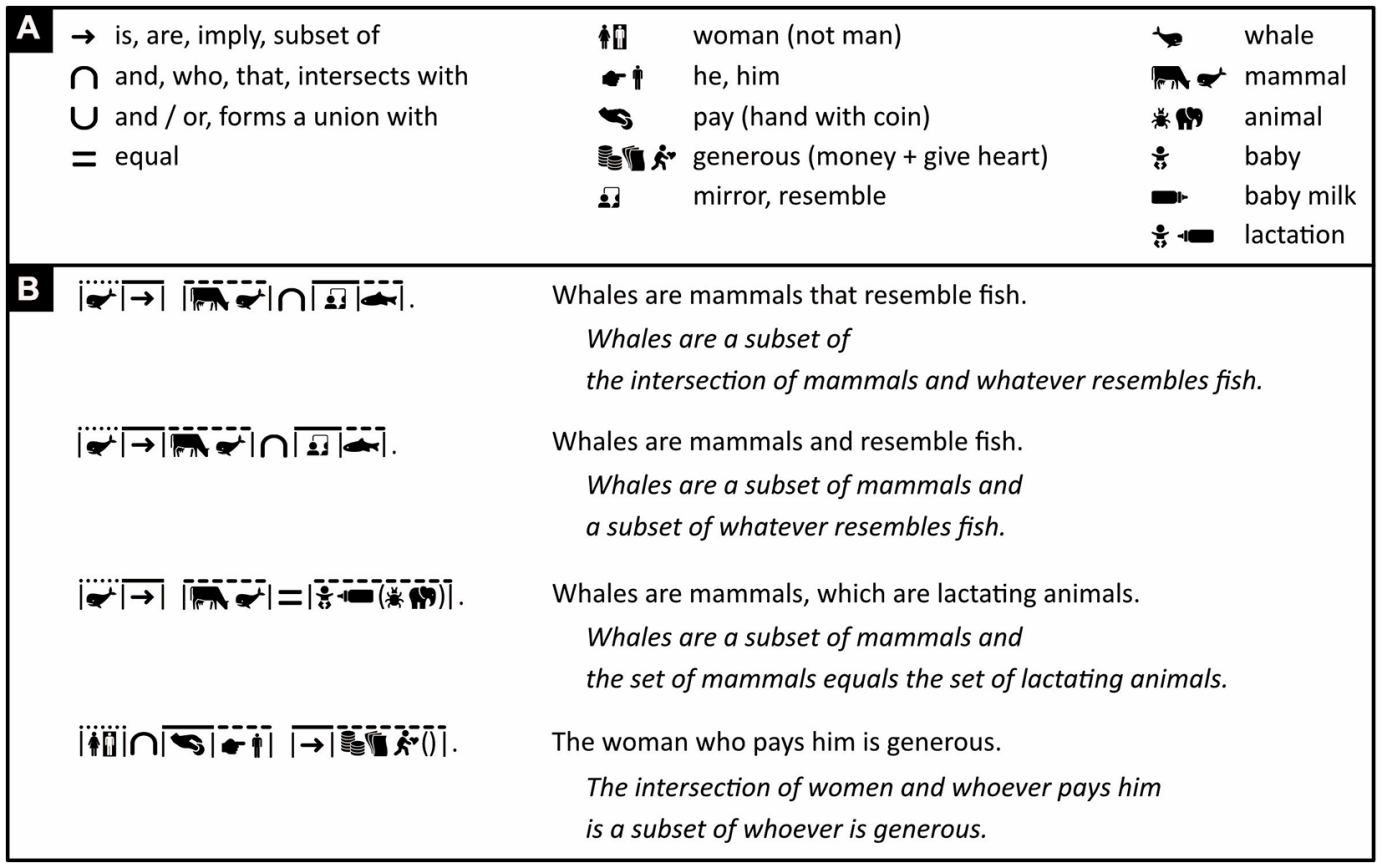
Active sentences with subjectless subclauses. **(A)** Iconic vocabulary used in the panel below, plus some associated words. (In Figure 6, “animal” was defined with the icons of a cow and a bird. Here, a synonym is used: the icons of an insect and an elephant) **(B)** Active sentences, as opposed to passive ones, containing a subclause without a subject. For each sentence in Icono, a loose English translation is provided, and in italics, a literal one. In Icono, the overall structure of complex sentences is clarified by either spaces between coherent passages or the omission of line segments above words that connect these passages, words like “who,” “that,” “and,” “equal.” Notice the subtle difference in meaning between lines 1 and 2, and notice that the subclause in line 3, unlike those elsewhere in this figure, does not restrict the meaning of the main clause. (Icons reproduced with permission from thenounproject.com; acknowledgments and links: https://osf.io/yrb49/).

**Figure 11:**
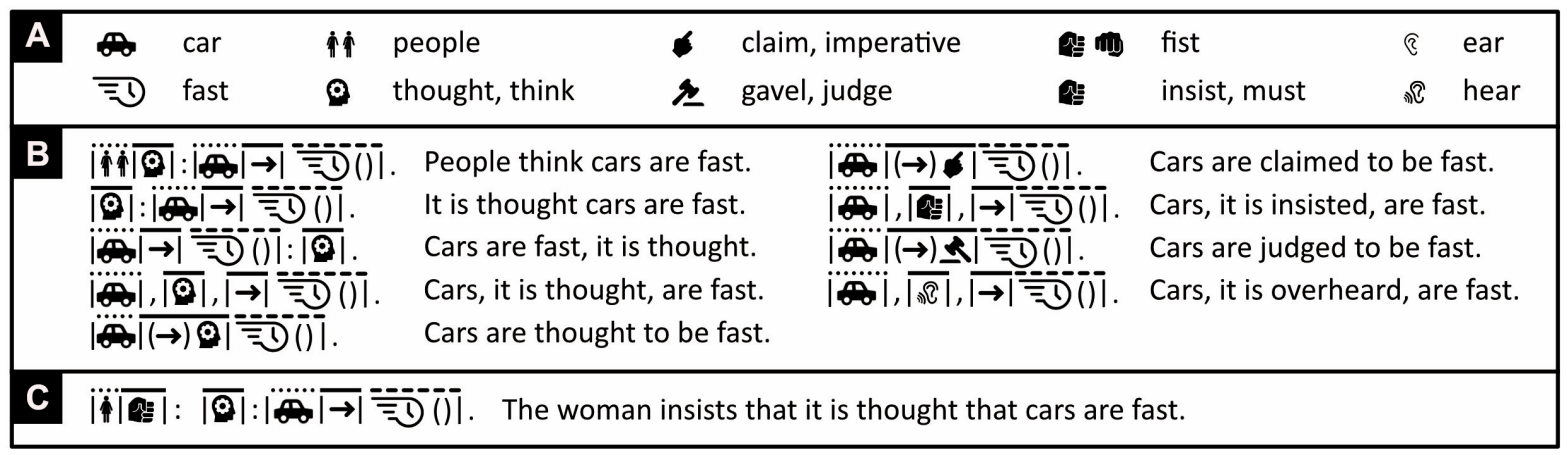
Passive sentences with subjectless subclauses. **(A)** Iconic vocabulary used in the panel below, plus some associated words. **(B)** An active sentence in Icono with a subclause (line 1, left) and various passive ones with a subclause (lines 2–4, left; lines 2 and 4, right) or without one (line 5, left; lines 1 and 3, right). Punctuation clarifies the overall structure of the sentences. Almost meaningless “dummy subjects,” like “*it* is thought,” are omitted. **(C)** A sentence with a subclause that, in turn, contains another subclause. To aid the correct parsing of the sentence, a little bit of space is added after the iconic translation of “the woman insists.” (Icons reproduced with permission from thenounproject.com; acknowledgments and links: https://osf.io/yrb49/).

**Figure 12:**
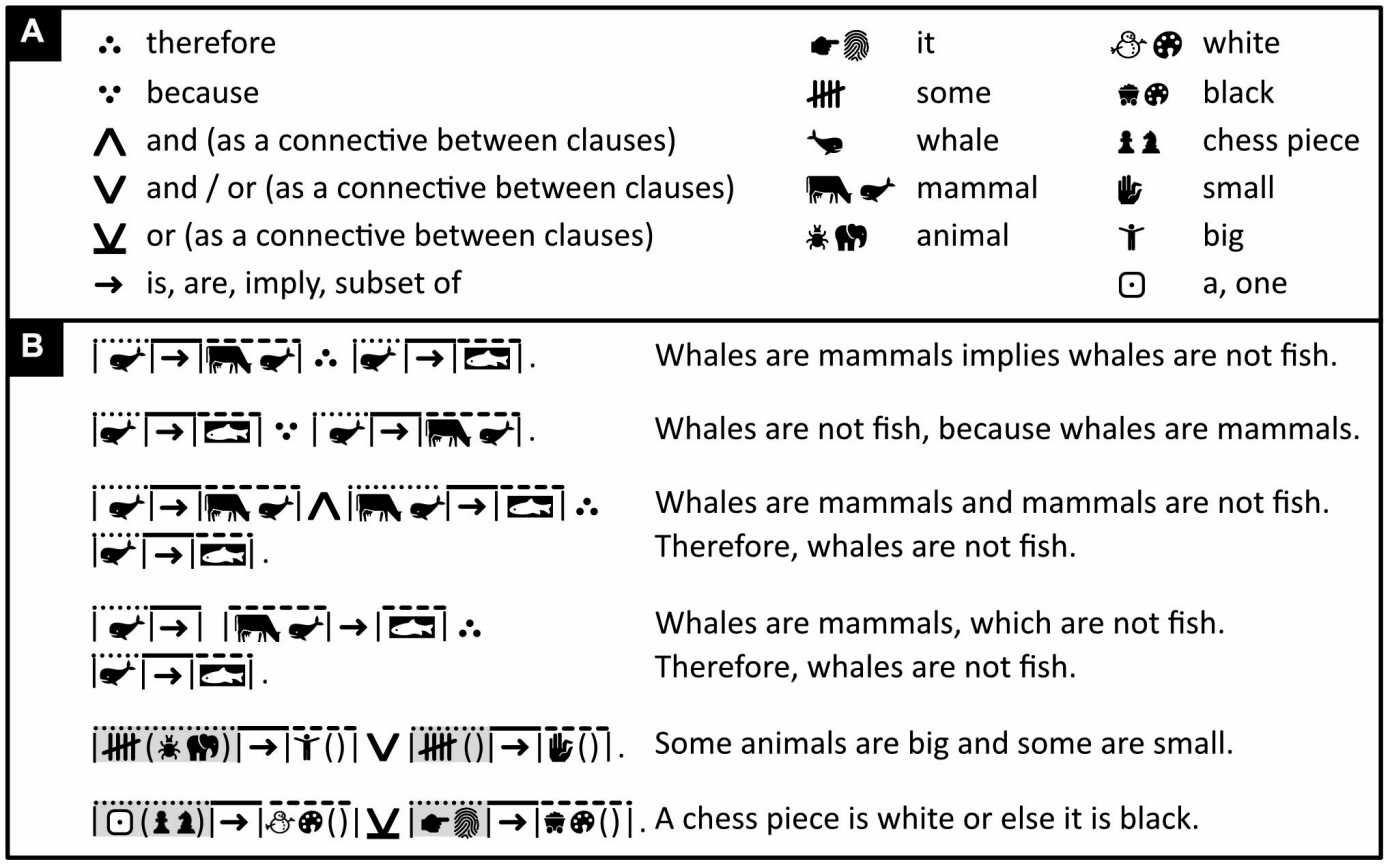
Sentences containing smaller sentences as subclauses. **(A)** Iconic vocabulary used in the panel below, plus some associated words. **(B)** Sentences containing a subclause that has its own subject, and in the fourth sentence, an additional subclause without a subject. Highlighting can be used to help the reader connect words that refer to the same thing. (Icons reproduced with permission from thenounproject.com; acknowledgments and links: https://osf.io/yrb49/).

Apparently, the Amazonian tribal language Pirahã avoids embedding simple sentences within complex ones, or more precisely, subclauses within main clauses (Futrell et al., 2016). Like almost all other languages, Icono does allow such iterative nesting. Icono’s subclauses may either lack (Figures 10B, 11B,C) or include (Figure 12B) an explicitly stated subject, and may either restrict (Figure 10B, lines 1, 2, 4) or leave unaffected (Figure 10B, line 3) the meaning of the main clause. To aid parsing, Icono uses punctuation, inserts some space between distinct passages, or separates such passages graphically by omitting grammatical markers above the words that connect these passages, words like “and,” “or,” “therefore,” “because.”

To avoid clutter, only the structure of subclauses is marked, not that of the sentence that contains these subclauses. For example, in “The woman who pays him is generous,” “The woman” is marked as a subject (with a dotted line segment), “pays” as a verb (with a continuous line segment), and “him” as an object (with a dashed line segment). “The woman who pays him” in its entirety, however, is not marked as the overarching sentence’s subject. To nonetheless ensure the correct parsing of this overarching sentence, its main subject must, by convention, always come before its main object—a constraint generally observed in natural languages as well.

As mentioned, words like “and,” “or,” “implies” can express relationships between sets. These same words can also be used to connect independent clauses to one another. To avoid ambiguity, Icono reserves different logical symbols for these two usages (compare Figure 12 to previous figures).

#### 2.2.8. Being deliberately obscure, vague, and indirect

**Figure 13:**
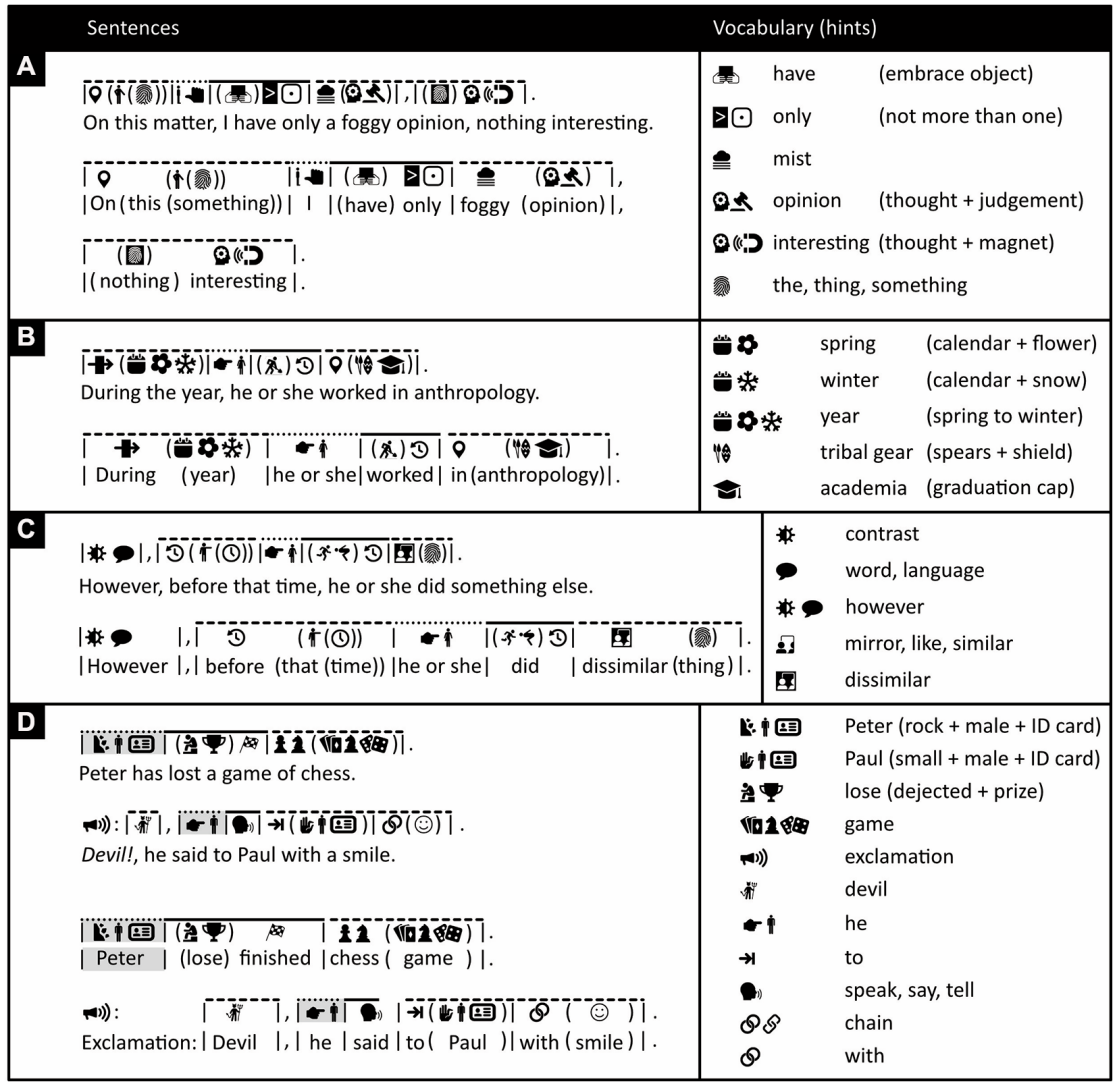
Using obscure language. **(A–D)** Obscure language (left) and associated vocabulary (right; for additional relevant vocabulary, see Figures 2, 3). In each panel on the left, an iconic sentence is presented along with its English translation, and then, within the same panel, it is repeated with the iconic and English words matched up closely for easier comparison. **(D)** This passage, composed of two interrelated sentences, exemplifies two figures of speech: hyperbole (Peter accuses Paul of being a devil) and irony (Peter accuses Paul while smiling). The passage also contains an example of “implicature”: it is not explicitly said, only implied, that Peter played chess against Paul. Icono, incidentally, highlights in gray that “Peter” and “he” have the same referent. (Icons reproduced with permission from thenounproject.com; acknowledgments and links: https://osf.io/yrb49/).

To communicate effectively and efficiently, it is usually wise to keep one’s sentences simple, concrete, and precise, and Icono is particularly well suited for this goal. Still, not everything can be expressed in concrete terms; being precise about details can detract from a big picture; the same material can sometimes be expressed more succinctly with a single complex sentence than with several simpler ones; or, for social reasons, some may prefer to avoid being upfront and to the point. Figures 10, 11, and 12 show how one can construct complex sentences in Icono; Figure 13 how one can make them obscure, abstract, vague, or indirect.

## 3. Technical issues

For the adoption of Icono to become practical, a few technical requirements need to be satisfied, none of them out of reach with existing means.

### 3.1. Building vocabulary

Icons have primarily been designed for commercial purposes or informal messages. New ones will be necessary to meet the needs in legal, literary, and academic communication. All icons need to be available in the public domain and, like emojis, should maintain their legibility when scaled down to approximately text size.

### 3.2. Creating a dictionary

An electronic dictionary is needed that translates English and other languages into Icono and vice versa. The translation of Icono into alphabetic languages is not as straightforward as the converse, because—like Chinese—Icono is not an alphabetic language, and its vocabulary can therefore not be alphabetically organized. Devices are already available, however, that scan Chinese text and translate it into other languages.^8^ Similar technology would come in handy for Icono.

### 3.3. Writing icons by entering alphabetic words

A popular and very fast way to write Chinese characters on electronic devices featuring regular Qwerty keyboards is to choose Chinese as one’s language and start typing in Pinyin. As soon as anything is entered, the app presents a list of characters consistent with the writer’s input in order of frequency of use. The writer can already select the sought-after character from the list, which inserts this character immediately into the text. Alternatively, the writer can add more input to shorten the list or provide input for two characters, rather than one, to get a very short list for their combination. Typically, with these options, words consisting of characters are written faster than they can be spelled out in Pinyin, let alone English (Mullaney, 2017).

**Figure 14:**
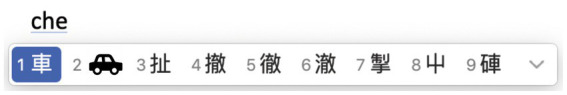
Writing icons by entering alphabetic words. What is shown here is what pops up on the screen if one uses a recent version of Apple’s operating system, chooses Chinese as one’s language, and writes “che” in Microsoft Word. The pop-up list consists of Chinese characters consistent with the pronunciation “che” plus a car emoji—in Chinese, “car” is pronounced as “che.” By typing in “2” right after “che,” what is inserted into the document is not “che2” but the second item of the list: a car emoji (here a car icon). The list of options then disappears, and the next character or icon can be written. (The car icon is reproduced with permission from the noun project.com; acknowledgments and links: https://osf.io/yrb49/).

Recent versions of Apple’s operating systems allow the use of Pinyin to write not only Chinese characters but also emojis (Figure 14). If the Chinese characters and emojis were replaced with Icono’s icons, such software could be used to write in Icono (Figure 14). As its input, this software should accept not only Pinyin but also words from the writer’s own language. Receiving “mouse” or “elephant,” for example, the app could immediately insert its iconic translation into the text. Receiving “mammal,” it would instead present a list of possible iconic translations, including the combination of the icons of a mouse and an elephant, and insert the one the writer selects into the text. The software should offer both black-on-white icons and white-on-black ones, and enable the addition of Icono’s grammatical marks (dotted, continuous, and dashed lines and such).

### 3.4. Writing icons by selecting word categories and subcategories

Writing Chinese characters does not necessarily require Pinyin or any other pronunciation-based script. One can use the *Cangjie* writing system, for example. Cangjie links character components to keyboard keys, and writers can assemble characters by pressing appropriate key sequences. Cangjie is harder to learn than Pinyin, but once learned, it enables even faster writing (Fong and Minett, 2012).

**Figure 15:**
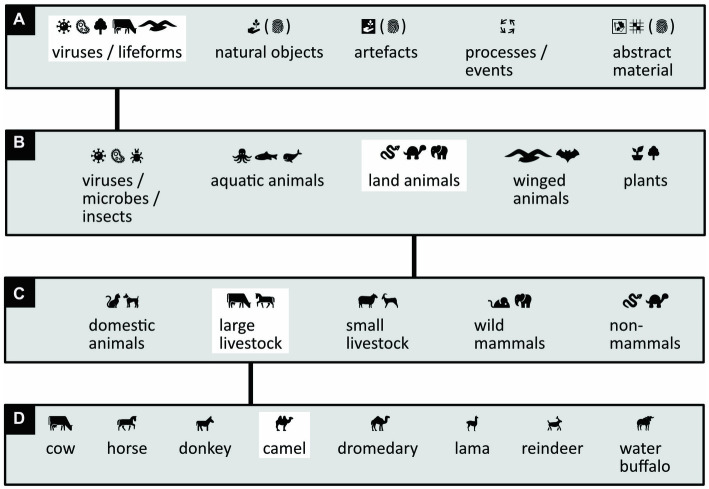
Writing icons by selecting word categories and subcategories. **(A–D)** Steps to go through to write the iconic version of the word “camel.” **(A)** Labels of icon-categories are shown to the writer (English translations are omitted), and the writer selects one of the categories (here highlighted in white). (The word “abstract” is visualized here with two icons of abstract paintings.) **(B)** Subcategory labels appear, and the writer makes another selection. **(C)** Subsubcategory labels appear, and the writer makes yet another selection. **(D)** Now the icons appear that the writer can select and directly insert in the text, like for example the “camel” icon. (Icons reproduced with permission from thenounproject.com; acknowledgments and links: https://osf.io/yrb49/).

Similarly, writing Icono need not necessarily require Pinyin, English script, or any other pronunciation-based script either. In principle, one could use a modified version of an app that is currently in use to help people communicate who have difficulty speaking, writing, and reading (Mirenda and Iacono, 2009; Ganz et al., 2012; Martínez-Santiago et al., 2018; see also Almurashi et al., 2022). For purely practical reasons, these apps organize words into categories and subcategories (for philosophical reasons to do something similar, see Weilgart, 1979; Okrent, 2010; Quijada, 2011; Lang, 2014; Wilkins, 2016). The writer selects a category by touching its pictorial label; subcategories appear, and the writer selects one; subsubcategories appear, and the writer selects one again; and finally pictorial words appear, in order of frequency of use, and the writer makes a final selection (Figure 15).

Suppose a vocabulary is built out of 1,000 different icons (with two icons per word, enough to let Icono rival Chinese), and suppose that these icons are organized into 5 categories that each contain 5 subcategories that, in turn, contain 5 subsubcategories (Figure 15): then, the final subsubcategory would contain on average 1,000/5/5/5 = 8 icons. To write out one of them, the reader would thus need to make three category selections (e.g., by typing in a number between 1 and 5 three times) plus a final selection of one icon out of eight (e.g., by typing a number between 1 and 8). Writing in this way would thus only require a numerical keyboard and no knowledge of any language’s spelling or pronunciation.

If the available set of icons is limited to 1,000, some words that could in principle be expressed with a single icon must nonetheless be expressed with more than one. For example, although yaks are not small, they are not listed among the large livestock in Figure 15. Hence, to write “yak” one has to use more than one icon—say, an icon of a cow plus an icon of a mountain. The alternative, of course, is to make more than 1,000 icons available. Doing so could force writers to make more category selections for each icon they wish to write. In compensation, however, the larger choice of icons would reduce the average number of icons needed per word.

## 4. Users with special needs

### 4.1. Dyslexia

Roughly 5 to 10% of the population is unusually poor in reading despite adequate schooling and having otherwise normal cognitive and perceptual abilities (Shaywitz et al., 1990; Peterson and Pennington, 2012; Ullman et al., 2020; Wagner et al., 2020). Such *dyslexia* comes in various degrees of severity (Wagner et al., 2020) and may have various unrelated causes but is most often associated with deficits in relating tokens to sounds and in learning and understanding grammar and parsing sentences (Melby-Lervåg et al., 2012; Peterson and Pennington, 2012; Huettig et al., 2018; Ullman et al., 2020; West et al., 2021; Li et al., 2022).

Icono is arguably better suited to dyslexics than are most other languages. First, dyslexics have trouble naming pictures (Faust et al., 2003) but no trouble recognizing what is depicted by either icons (Berget et al., 2016) or pictures (Faust et al., 2003; Greenham et al., 2003; Hanly and Vandenberg, 2010). Second, dyslexics have much more trouble naming a picture of, say, a pineapple than linking this picture to a different but semantically related picture of, say, a pair of cherries rather than a pair of glasses (Johnson et al., 1996; Araújo et al., 2016; see also Jednoróg et al., 2010; Jones et al., 2016). This suggests that dyslexics not only have no trouble recognizing what pictures depict but also no trouble categorizing them correctly as, say, instances of fruit. Third, unlike in other languages, Icono’s sentence structure is visible before one even begins reading, and this should make parsing sentences easier for everyone, including dyslexics. And fourth, unlike most other languages, Icono does not require that one relates tokens to sounds or sounds to meanings, and hence it is unaffected by the typical deficits dyslexics have in this domain (Reis et al., 2020).

The ability of dyslexics to spontaneously pick up a grammar and make it their own is sometimes investigated with strings of letters or shapes whose order is constrained by an undisclosed set of rules—an *artificial grammar* (for a meta-analysis, see Van Witteloostuijn et al., 2017). Most dyslexia research, however, focuses on dyslexics’ ability to process natural grammars and natural language. It is typically assumed that dyslexia is a personal problem of unfortunate individuals. Partly, however, it is also a problem created by a society that uses a language that is unfriendly to readers. The opaque spelling of English, for example, has repeatedly been shown to hinder learning to read (Ziegler and Goswami, 2005; Share, 2008; Caravolas et al., 2013) and to exacerbate dyslexia (Landerl et al., 2013; Lallier et al., 2018; Reis et al., 2020). And yet, to my knowledge, not a single study has investigated whether dyslexics might find it easier to learn to read, say, Esperanto rather than their own natural, but irregular, language.

In any case, because iconic words engage the brain very differently than do alphabetic words or even Chinese characters, it might be a good idea to investigate whether dyslexics could have less trouble reading an icon-based artificial language than an alphabetic or character-based natural one. That this might indeed be the case is suggested by research that shows that, more than others, dyslexics seek to form a mental image of alphabetic words (Evans et al., 2022) and have less trouble reading icons than reading alphabetic words (Berget et al., 2016). That is, while searching for a verbally communicated target item within a grid or list of items, dyslexics are slower than non-dyslexics when these items contain written words but not when they only contain icons. And whereas reading alphabetic traffic warnings impair concurrent simulated driving more in dyslexics than in non-dyslexics, recognizing iconic road signs with the same message does not (Roca et al., 2018). These findings suggest that dyslexics may perhaps find an icon-based language like Icono easier to read than an alphabetic one. Modern technology can convert text into speech and speech into text. Yet speech is fleeting in a way text is not. Hence, especially in case of complex formulations or subject matters, dyslexics may often still prefer to read and write rather than to listen and speak.

### 4.2. Stroke, autism, cerebral palsy, cognitive impairment, and Alzheimer’s

Imagine you would like to ask for some water but have cerebral palsy and thus poor control over muscles needed for speaking, gesturing, and writing. Or imagine you know what you want and have the muscle-control to express this, but a stroke and resulting aphasia have selectively damaged the language centers in your brain, blocking you from communicating effectively. Or imagine that because of mild cognitive impairment or Alzheimer’s you keep forgetting what words mean but can still recognize pictures. Or imagine that, because of severe autism, you can express yourself with little else than temper tantrums. Some evidence suggests that in such cases pictorial “languages” like Picture Exchange Communication System and Blissymbolics can help bypass the deficits and perhaps even ameliorate them a little (Koul et al., 2005; Rajaram et al., 2012; Gilroy et al., 2017; Brignell et al., 2018; Sevcik et al., 2018; Almurashi et al., 2022; see also Cherry et al., 2008; Ally et al., 2009; Embree et al., 2012; but *cf.* Meza et al., 2022). Typically, however, these languages use pictures that are either more cluttered than necessary, like in the case of PECS, or more abstract and symbol-like than necessary, like in the case of Blissymbolics (Mizuko, 1987; Alant et al., 2005). Icono’s icons, instead, avoid both unnecessary clutter and unnecessary abstraction; they concretely depict only the gist of concepts and, compared to their equivalents in PECS and Blissymbolics, should therefore be easier to read, understand, learn, and use (Bühler, 2021).

## 5. Discussion: how can Icono succeed where Esperanto failed

### 5.1. Esperanto without hope

Esperanto, created by L.L. Zamenhof (Zamenhof, 1909), is by far the most popular artificial language today, and by design, it is much easier to learn and use than English or any other natural language (Garvía and Soto, 2015; Lins, 2017; Goodall, 2023). Like English, it uses the Roman alphabet, but unlike English, its spelling is transparent rather than opaque and its grammar sophisticated but regular and simple rather than irregular and needlessly complex. Esperanto borrows its vocabulary exclusively from European languages (mainly Latin ones) and its grammar mostly from Indo-European ones. Like the Turkish and Finno-Ugric languages, however, Esperanto does often “glue” meaningful additions to existing words (agglutination).

“Esperanto” derives from the Latin “sperare” (to hope). Intended was not so much hope for the language itself but hope it could bring people together—at least on speaking terms. Unfortunately, hope for Esperanto has been in short supply. In the 1920s, the language did gain modest popularity, especially in Europe but also beyond (Garvía and Soto, 2015; Lins, 2017; Goodall, 2023). At its peak, Esperanto was promoted through some 60 Esperanto periodicals worldwide and some 1,300 Esperanto clubs; the language was an elective or mandatory subject in several schools in several different countries, including the United Kingdom; and some children of Esperantists even grew up with Esperanto as their mother tongue (Garvía and Soto, 2015; Lins, 2017). Yet, in the 1920s, the French Minister of Public Instruction, Léon Bérard, promoted French as a world language and prohibited the teaching of Esperanto in French schools (Garvía and Soto, 2015; Lins, 2017). Bérard later became an ambassador for Vichy France, which had a Nazi friendly regime, and Esperanto just so happened to be invented by a Jew (from Belostok, Russian Empire, now Białystok Poland). Indeed, Hitler and Stalin and several of their allies shut down all Esperanto organizations under their control and persecuted, and in some cases executed, these organizations’ most prominent leaders (Garvía and Soto, 2015; Lins, 2017).

Eventually Anglo-Saxon economic, cultural, and military expansion pushed French to the side and English to the fore (Garvía and Soto, 2015; Lins, 2017). Esperanto was left behind and never gained the political backing that the broad acceptance of impactful linguistic innovations typically requires (Fischer, 2001). English is now the world language, but not because it is more suitable for this task than Esperanto. Indeed, English is clearly far too hard for far too many. English proficiency among non-native speakers is deemed to be high in only 31 out of 112 countries, and tellingly, in many of these 31, people’s native language is closely related to English.^9^ Only three Asian countries perform well: Malaysia and Singapore, both former British colonies, and the Philippines, a former American colony. Even many of the world’s most educated scholars and scientists, with access to translation software and spelling and grammar checkers, still need help with their English. Indeed, English editing services are so much in demand that many scientific journals have deemed it best to partner with them.

Language can be used not only to communicate but also to bamboozle, divide, or dominate other people (Fischer, 2001). This may be why bureaucrats, lawyers, and scientists use more jargon than necessary, why medical doctors appear to offer a diagnosis when merely labeling signs and symptoms in Greek or Latin, and indeed why the world language is English rather than a language that is independent of any economic, political, or military power. Facilitating communication is also hardly the reason why Catholic Croats and Orthodox Serbs embrace different scripts for their joint language of Serbo-Croatian or why Hindu Indians and Muslim Indians and Pakistani do so for their joint language of Hindustani (Hindi/Urdu).

Against the odds, the League of Nations once did come close to adopting Esperanto. Partly due to French intervention, however, the idea was eventually sidelined (Garvía and Soto, 2015). UNESCO’s 1954 Montevideo Resolution officially recognizes Esperanto as an international auxiliary language (Resolution IV.4.422–4,224). Yet neither the United Nations nor the European Union have adopted, as an official language, either Esperanto or any other language specifically designed to make international communication easy, nor do they promote the development of any such language.

### 5.2. Hope without Esperanto

Motivating people to learn a new language that is not guaranteed to ever become widely used is a challenge. The official adoption of a new language by a nation or large organization is therefore of major help. This is, in fact, how Hebrew was brought back from the dead (Okrent, 2010). Lack of political support may thus go a long way to explain why neither Esperanto nor any of its rivals ever became a success. Yet the fact that none of these languages are as user-friendly as they could have been may very well be the most important reason for their failure.

Six interrelated reasons suggest that Icono is more engaging than Esperanto and much easier to learn, and should therefore stand a better chance of success. First, in Icono, unlike in both English and Esperanto, most words illustrate their own meaning and are therefore inherently reader-friendly and inherently international. Second, in Icono, unlike in both English and Esperanto, most words consist of icons, and these are more engaging and easier to interpret than their alphabetic equivalents. Third, in Icono, unlike in both English and Esperanto, sentences reveal their structure when it is most helpful: before, rather than after, one begins reading. Fourth, Icono, unlike both English and Esperanto, is not extra hard on the half of the world’s population whose native language is not Indo-European^10^ and may be of help to people with conditions like dyslexia, aphasia, cerebral palsy, and autism with speech impairment. Fifth, learning to read or write in Icono, unlike in English or Esperanto, does not require one to memorize the arbitrary associations between the meaning of words and their pronunciation or spelling. And sixth, on smart phones and computers, one can write iconic words faster than one can spell out alphabetic ones.

### 5.3. Response to common objections

#### 5.3.1. Icono cannot beat language imperialism

Some would have it that when the hegemony of English as the world language ends, there will simply be a new political power that imposes its language on us all. Consider, however, that the world did let utility triumph over politics when it adopted one single enduring universal language in which to conduct mathematics and logic. Potentially crucial in its success may have been that this language, unlike English and Esperanto, but just like Icono, does not impose any particular pronunciation and does not twist anyone’s tongue.

#### 5.3.2. Icono cannot beat translation software

Some would have it that there is no need for an efficient universal language anymore, as translation software can help us out in international correspondence. Consider, however, that even among the world’s most educated, with access to very helpful software, English editing services are still in high demand. This suggest that current translation software falls far short of what is needed.

#### 5.3.3. Icono is dead on arrival

Some would have it that artificial universal languages have always been dead on arrival and always will be. Indeed, in linguistics, even the mere study of artificial languages is frowned upon (Okrent, 2010). Consider, however, that it was not a lack of success that caused Esperanto’s demise but the very opposite. Esperanto did not die a natural death: it was killed. Now that the assassins are gone, however, there is no need for self-fulfilling fatalism.

#### 5.3.4. Icono, if adopted, will become a mess

Some would have it that an artificial language, once adopted, will inevitably become irregular and messy itself, just like all natural ones always have (Okrent, 2010). Consider, however, that given sufficient political will, the very opposite can also be achieved (Fischer, 2001; Coulmas, 2003). This is why Turkish, for example, now has a transparent script that can express all its vowels rather than just a few—courtesy of Mustafa Kemal, “father of the Turks.” And this is also why, each year, both North and South Koreans celebrate Korean Alphabet Day, reminding themselves of the fact that they now have the world’s first and only iconic alphabet—courtesy of king Sejong the Great (see Supplementary material).

#### 5.3.5. Icono’s concrete icons can never express abstract concepts

Some would have it that an iconic word can never express an abstract concept because it is always tied to a concrete image (Lupyan and Winter, 2018). Consider, however, that in Icono one can for example use the icon of a bird plus the icon of a rocket to express not “fly like a bird” or “fly like a rocket” but “fly” in the abstract. Likewise, partly thanks to ancient Chinese scribes and especially modern icon designers (see thenounproject.com), it is now possible to visualize numerous concepts one might previously have deemed too abstract to depict, like in this article the concepts of “use,” “lie,” “may,” “might,” “adverb,” “past tense,” “year,” “opinion,” “interesting,” “anthropology,” “however,” and many others.

#### 5.3.6. Icono’s concrete icons are unhelpful to readers

Some would have it that iconic words are unhelpful to readers, because they are more easily confused with one another than are non-iconic ones (Lupyan and Winter, 2018). “Iconic” is meant very broadly by these critics, and words whose sound reminds one of their meaning, for example, are also deemed iconic. Consider, however, that such metaphorical icons typically do not hint at their meaning as clearly as real ones do; that metaphorically iconic words typically contain at most one icon, whereas Icono’s words often contain two or more (Section 2.1.1); and that metaphorically iconic words contain only one type of icons, whereas Icono’s words can contain both positive black-on-white icons and negative white-on-black ones (Section 2.1.2).

Onomatopoeic words like “dripple,” “giggle,” or “clap,” for example, are metaphorically iconic, and each consists of only one auditory hint to its meaning—one auditory “icon.” Other spoken words are weakly iconic at best. For example, across languages, words for l*a*rge things and t*ee*n*y* w*ee*n*y* ones appear to disproportionately often contain, respectively, an /a/ or an /i/ sound. Because languages are not mutually independent, however, the effect might not be genuine. Likewise, in Sign Language, words are not seldom metaphorically iconic too. The sign for “eat,” for example, is commonly depicted by fingers moving toward the mouth, which is quite intuitive. Signed words, however, typically consist of just one sign and thus at most one metaphorical icon. Signs can be combined, but in this process, signers typically cut corners, compress and merge the signs, and make them less rather than more transparent (Sandler et al., 2006).

In natural languages, the only written words that are somewhat iconic are Chinese. Each Chinese character typically features several pictograms or ideograms. As they became easier to write, however, these *radicals* became much less iconic (Figure 1; Xiao and Treiman, 2012). Many of them, moreover, are not reliable hints to a character’s meaning, but unreliable hints to its pronunciation (Sections 1.1 and 1.3). Worse, as they come from a fixed set of 214, these radicals are often not the most suitable ones with which to express a word and their combinations can be opaque and overly complex (Sections 2.1.2 and 2.1.3). Indeed, Chinese characters are notoriously difficult to learn, and it is hardly a surprise that their remaining iconicity is at best of little help (Yum et al., 2011; Lo and Yeh, 2018).

Interestingly, the Chinese word for “mammal,” 哺乳動物, is its own definition and means “breast-feeding animal” (Section 2.1.2). Yet it consists of almost a dozen pictograms and ideograms, their depictions are hard to recognize, and only some of them are direct hints to the word’s meaning (the others are rough hints to its pronunciation). To express the same concept, Icono typically uses just two highly recognizable icons: one of a cow and one of a mouse—their meaning given by what they have in common (Figure 2). Icono offers key mnemonics to the meaning of “mammal,” not a full-fledged definition of it (Section 2.1.2). Tellingly, indeed, people who understand something are not said to get the definition: they are said to *get the picture* and to *see what one is saying*.

### 5.4. Final thoughts

Those who call a train “一列火車,” “ein Zug,” or “أو قطار واحد” may not understand each other, and to many people, Esperanto’s “unu trajno” and the English “a train” are just as unintelligible. Yet all of us, illiterates included, instantly understand what a little picture of a train represents. Likewise, for many, deciphering 四, ٤, or Դ is a challenge, but that four dots could mean “4” is not hard to wrap one’s brain around. And although the icon of a magnifying glass on a person’s skull may not remind everyone of “psychology” (people getting their heads examined), learning this association is far from a challenge. Only in those exceptional cases in which it *completely* fails to visualize the meaning of a word does Icono become as unhelpful to readers and language learners as English and Esperanto are to Arabs and Chinese.

At no cost to the writer (thanks to modern technology), Icono puts readers first. Even fluent readers understand, recognize, and recall better and faster what simple pictures depict naturally than what corresponding strings of letters mean conventionally. Icono’s building blocks are therefore not arbitrary squiggles that can only be read by those in the know but carefully chosen icons that are easy for the brain to process; that illustrate, and help readers recall, what they stand for; and that are accessible to readers across the world—arguably even to some who struggle with ordinary texts. These icons, moreover, can express not only concrete concepts but highly abstract ones as well. Icono’s sentence structure, meanwhile, is revealed when most helpful: before, rather than after, one begins reading. Because learning its pronunciation or phonetic spelling is optional rather than a prerequisite, and because it shows what it says, Icono is bound to be easier to learn to read—and then easier to read—than any other language, including our own.

## Author contributions

The author confirms being the sole contributor of this work and has approved it for publication.

## Conflict of interest

The author declares that the research was conducted in the absence of any commercial or financial relationships that could be construed as a potential conflict of interest.

## Publisher’s note

All claims expressed in this article are solely those of the authors and do not necessarily represent those of their affiliated organizations, or those of the publisher, the editors and the reviewers. Any product that may be evaluated in this article, or claim that may be made by its manufacturer, is not guaranteed or endorsed by the publisher.

## Footnotes

^1^The Dongba script of the Chinese Naxi people is pictorial but of limited, mostly religious, use (Nie and Yao, 2022).

^2^In the present article, because they are a little closer to their pictorial origin, traditional rather than simplified characters are used, like in Taiwan, Hong-Kong, Macao, and Japan.

^3^thenounproject.com

^4^http://www.iconji.com/

^5^www.blissymbolics.org

^6^https://osf.io/yrb49/

^7^https://thenounproject.com

^8^http://www.waygoapp.com/

^9^www.ef.com/epi

^10^www.ethnologue.com/guides/largest-families
