# Supplementary material for: Icono: a universal language that shows what it says
Source: Front Psychol. 2023 Jul 28;14:1149381. doi: 10.3389/fpsyg.2023.1149381 (PMC10421668; doi:10.3389/fpsyg.2023.1149381)
Supplement: Supplementary file 1 [file Data_Sheet_1.pdf]

Supplementary Material

Icono: A Universal Language that Shows What It Says

Icono's Universal Pronunciation

Peter Kramer

Department of General Psychology, University of Padua, Padua, Italy

Correspondence:

orcid.org/0000-0003-4807-7077

peter.kramer@unipd.it

Like mathematics, Icono can be pronounced in any language. For international communication, however, it would ideally be pronounced in just one, universal language. In principle, English—the current world language—can be used for this purpose; yet, for many, English is hard to pronounce, and for this reason, Icono has a pronunciation of its own that is specifically designed to be easy for all peoples of the world. Icono assigns an exceedingly simple pronunciation to each icon, and the pronunciation of Icono's words is dictated by the pronunciation of each of these words' constituent icons. As a result, words that have icons in common also have a part of their pronunciation in common. Unlike written ones, however, spoken words can only offer few and rather weak iconic hints to their own meaning (see main article Section 5.3.6). Inevitably, therefore, Icono is considerably easier to learn to write than to learn to speak. Still, if we wish to have a world language everyone can pronounce, Section 1 describes how this can be achieved, and Section 2 introduces a script, inspired by Korea's *Hangul* script, that represents Icono's sounds in an intuitive, iconic way.

1 Words anyone can pronounce

Languages differ greatly regarding the sounds they treat as either distinct phonemes (the smallest units of speech that affect meaning: vowels and consonants) or indistinct allophones (variations in pronunciation that do not affect meaning). To accommodate everyone, Icono therefore adopts as phonemes only those sounds that are shared by virtually all languages of the world. Icono also treats as allophones all sounds that are easily confused with one another. Respecting these constraints, Icono ends up with just 12 phonemes. By comparison, the Papuan language of Rotokas, which

phonetically is regarded as the simplest of all natural languages (Robinson, 2006), comprises 14 different phonemes in its Aita dialect (five vowels and nine consonants) and 16 in its Central dialect (five vowels, each with a short and a long variant, and six consonants).

1.1 Icono's vowels

Among the easiest vowels to tell apart are the three phonetically transcribed as /i/, /u/, and /a/, respectively pronounced as in "m*ee*t", "b*oo*t", and "b*a*r", which are produced very differently and in distant parts of the mouth (Weckwerth, 2021; Ladefoged & Maddieson, 1996). Virtually all languages of the world distinguish these three vowels, and few people will find it hard to pronounce these vowels, /i/, /u/, and /a/, and their longer versions: /iː/, /uː/, /aː/. Modern standard Arabic, spoken by many millions of people, does not distinguish any more than these 3×2=6 vowels. Icono therefore also only adopts these six, which—with some leeway—are pronounced as: /i/, /u/, /a/, /iː/, /uː/, /aː/.

1.2 Icono's consonants

Of the consonants, the easiest to pronounce and globally most widespread are phonetically transcribed as /b/, /p/, /d/, /t/, /g/, /k/ (Ladefoged & Maddieson, 1996; Proctor, 2021). The pronunciations of /b/, /d/, /g/ are identical to those of respectively /p/, /t/, /k/ except that the former are voiced (make the vocal cords vibrate) whereas the latter are not. Voiced consonants are easily confused with their unvoiced counterparts, and some languages—including the Austronesian ones of Hawaiian and Maori—do not even distinguish the voiced from unvoiced ones. Icono therefore allows them to be used interchangeably as allophones of just three phonemes. The next globally most widespread consonants are transcribed as /n/ and /m/. Because they are easily confused, Icono treats them as two allophones of a single consonant. The final two consonants of Icono are /f/ and /s/, which are very common across languages as well. Icono thus ends up with a total of six consonants that, with some leeway, are pronounced as: /p/, /t/, /k/, /n/, /f/, /s/ or equivalently: /b/, /d/, /g/, /m/, /f/, /s/.

1.3 Icono's syllables

Chinese, Japanese, and various Austronesian and Papuan languages avoid the use of consonant clusters and limit writing or pronouncing a consonant at the end of a syllable. To accommodate the very large number of people who speak one these languages as their native tongue, each of Icono's icons is pronounced with one syllable consisting of one consonant followed by one vowel. Given that it has six vowels and six consonants, Icono thus ends up with 6×6=36 different syllables. It can therefore assign unique pronunciations to 36 one-syllable words, 36×36=1296 two-syllable words, 36×36×36=46656 three-syllable words, and so forth. The fewer distinct syllables a language has, the longer its words must be or the more homophones (identical-sounding words) and near-homophones (similar-sounding words) it must contain. Indeed, Austronesian languages tend to have long words and Chinese many homophones and near-homophones. To be concise and nonetheless unambiguous, Icono emulates a solution offered by the Chinese language.

1.4 Dealing with homophones

Chinese words tend to be very short: just one or two characters in writing, which corresponds to one or two syllables in speech. These short words are unique in writing, but many of them sound similar or identical in speech. To disambiguate them, these short words are often temporarily prolonged a

little. Color names, for example, tend to be just one syllable long and to sound similar or identical to various other words. Yet, if context does not disambiguate these names already, the word "color" (in Chinese expressed with just one syllable) is often appended to them to prevent confusion: "white" becomes "white color", "red" "red color", and so on. To disambiguate words, one can also temporarily append a synonym to them. Once the listener can be assumed to have understood which word is meant, the synonym can then be dropped again. Of course, solutions like these are only needed for short words, which are the most likely to have homonyms. Thus, whereas in most languages words have a fixed length, in Chinese and Icono they are as long as necessary and as short as circumstances allow.

Especially when simple words are combined into more complex ones, these simple ones can often remain very short without causing confusion. For example, in Chinese, the simple words of "fire", 火, and "partner", 夥, have different meanings but are both pronounced as *huǒ* (with *ǒ* pronounced as *o* with a briefly dipping intonation). Yet, within the compound word "fire vehicle", 火車, pronounced as *huǒ chē* (with the last letter having a flat intonation), *huǒ* can only mean "fire", 火, not "partner", 夥, and hence there is no need to disambiguate and prolong anything. Likewise, the Chinese word for "bus", 公車, translates as "public vehicle". Yet, in a conversation about busses, or standing near a bus stop, it is quite acceptable and common practice to transform 公車 into 車子, which is slightly faster to pronounce but means "vehicle". In principle, it could be abbreviated to just 車, which also means vehicle. Icono follows Chinese in abbreviating words whenever this does not confuse the listener (or reader).

2 Letters that illustrate their own pronunciation

English spelling is notoriously opaque, and English dictionaries and language-instruction material therefore typically use one alphabet for English words and another (*IPA*: *International Phonetic Alphabet*) to express the pronunciation of these words (Association & Press, 1999). Similarly, Icono uses one script that illustrates the meaning of words, henceforth called *Reader's Icono*, and another that illustrates the pronunciation of these words, henceforth called *Speaker's Icono*. Reader's Icono is discussed in the main article and was until now simply referred to as "Icono". Speaker's Icono is discussed here. Proficient readers of English rarely need IPA to know how English words are pronounced, and proficient readers of Reader's Icono will not need Speaker's Icono much either. Yet, for those who are learning to speak English, IPA is a great help, and likewise, for those who are learning to speak Icono, Speaker's Icono will be too.

IPA is used to clarify the pronunciation of many languages but is not particularly reader friendly; it consists of arbitrary squiggles whose pronunciations are merely a convention. The same goes for Pinyin in Chinese dictionaries. In contrast, the ingenious *Hangul* script that in Korea has now largely replaced Chinese characters does reach out to the reader: its consonants—although not its vowels—are written with tiny icons that, in a minimalistic way, illustrate how to use the mouth to pronounce these very consonants: ㄱ and ㄴ, for example, show how to curve the tongue inside the mouth to pronounce respectively the /k/ and /n/ sounds; ㅅ resembles a tooth and hints at the fact that to pronounce the /s/ sound one needs to involve the teeth (Fischer, 2001; https://en.wikipedia.org/wiki/Hangul). Speaker's Icono adopts this ergonomic idea for its own alphabet but uses icons that, although harder to write by hand, feature more recognizable hints to their own pronunciation (Supplementary Figure 1).

	Speaker's Icono	IPA	Comments
(A)	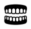	/i/	voiced with teeth exposed
	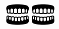	/iː/	long version
	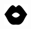	/u/	voiced with a protruding mouth
	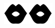	/uː/	long version
	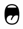	/a/	voiced with wide open mouth
	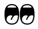	/aː/	long version
(B)	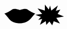	/p/	lips closed, followed by short burst of exhaled air
	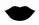	/p/	abbreviated
	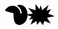	/k/	tongue curved down to lower teeth, followed by short burst of exhaled air
	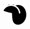	/k/	abbreviated
	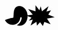	/t/	tongue curved up to upper teeth, followed by short burst of exhaled air
	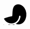	/t/	abbreviated
	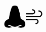	/n/	continuously voiced nasal sound
	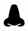	/n/	abbreviated
	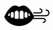	/f/	continuously unvoiced hissing with upper teeth on lower lip
	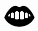	/f/	abbreviated
	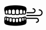	/s/	continuously unvoiced hissing with teeth exposed

Supplementary Figure 1: Speaker's Icono. All vowels (A) and consonants (B) of Speaker's Icono, along with their equivalents in the International Phonetic Alphabet (IPA), plus brief descriptions of how they are pronounced. (Icons reproduced with permission from thenounproject.com; acknowledgments and links: https://osf.io/yrb49/.)

Just like written Chinese tends to be considerably more concise than spoken Chinese, Reader's Icono is also considerably more concise than Speaker's Icono. For every icon in Reader's Icono, for example, Speaker's Icono must use at least two icons to express its pronunciation: one or two to represent its consonant and one or two to represent its vowel (Supplementary Figure 1). Moreover, Reader's Icono expresses its grammar with short vertical bars; horizontal dotted, continuous, and dashed lines; punctuation; and highlighting. At least some of these must have pronounceable iconic alternatives in Speaker's Icono. To this end, subjects, verbs, and objects in Speaker's Icono are each preceded by their own pronounceable iconic marker, and so are brackets (Supplementary Figure 2). Highlighting, however, is simply ignored. The vertical bars that distinguish words from one another are expressed with short pauses.

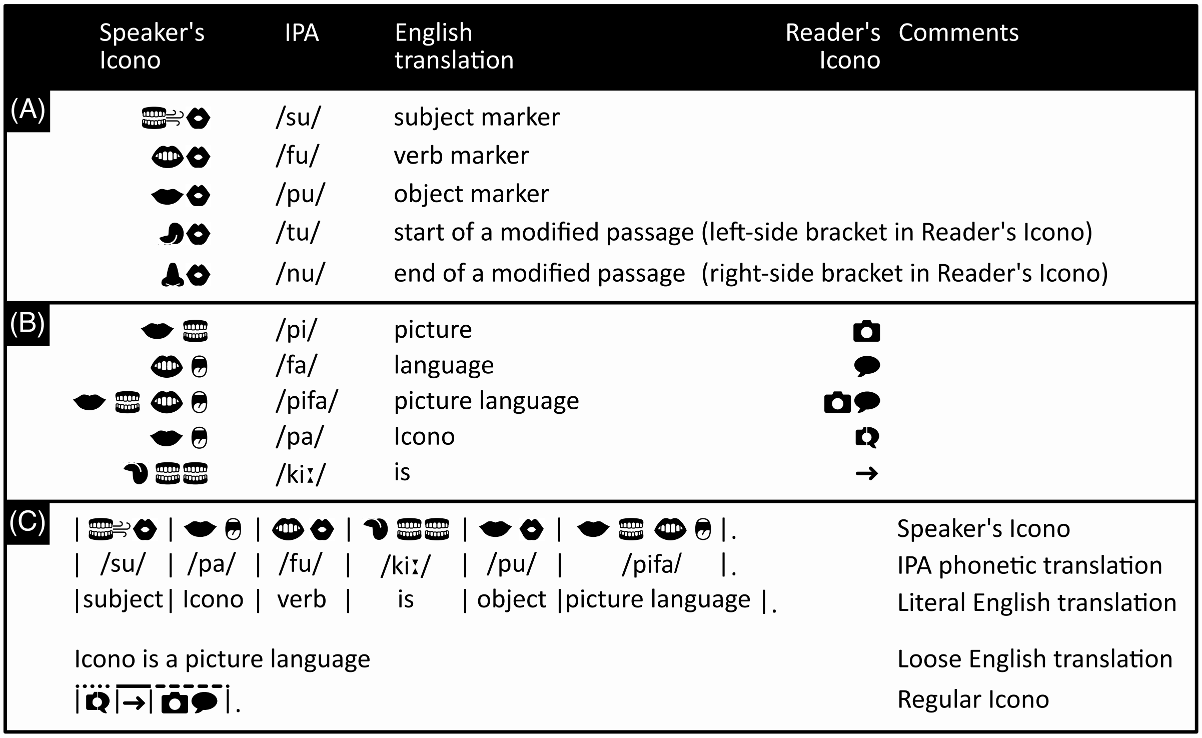

Supplementary Figure 2: Sentences in Speaker's Icono. (A) All five of Speaker's Icono's grammatical markers. (B) Some vocabulary in both Speaker's Icono (left) and Reader's Icono (right). (C) An exceedingly simple sentence in Speaker's Icono that uses three of Speaker's Icono's five grammatical markers. The sentence is followed by its transcription into the International Phonetic Alphabet (IPA); its literal, word-for-word English translation; its looser English translation; and its translation into Reader's Icono. (Icons reproduced with permission from thenounproject.com; acknowledgments and links: https://osf.io/yrb49/.)

3 The costs and benefits of a simple pronunciation

In more than a third of the world's languages, the meaning of a word depends on its intonation (Lee & Mok, 2021). In Chinese, for example, *mā* with a flat intonation means "mother", *má* with a rising one "hemp", *mǎ* with a briefly dipping one "horse", and *mà* with a falling one "scold"; *ma*, without much of an intonation, functions as a question mark. An important advantage of using intonation in this way is that it allows more words to be short and nonetheless distinct. In general, the more phonemes a language has, the more concise it can be. The artificial language of *Ithkuil* exploits this circumstance to the maximum extent possible (Quijada, 2011) and features not only intonation but also, among other things, Caucasian guttural sounds, Khoisan clicks, and Southern-Bantu whistled fricatives (see also Ladefoged & Maddieson, 1996; Proctor, 2021). It would be tempting to pronounce, say, Icono's black-on-white icons with a high intonation and white-on-black ones with a low one. Highly desirable would also be to help listeners segment speech by letting speakers stress the first syllable of each word, as is done in Finnish and Hungarian. For now, though, Icono keeps its pronunciation as simple as it can be. Keeping it simple helps make Icono's pronunciation less susceptible to change over time and across linguistic communities and this benefits not only speakers but listeners too.

4 References

Association, I. P., & Press, C. U. (1999). *Handbook of the International Phonetic Association*. Cambridge, UK: Cambridge University Press.

Fischer, S. R. (2001). *History of Writing*. London: Reaktion Books.

Ladefoged, P., & Maddieson, I. (1996). *The Sounds of the World's Languages*. Camrbridge, MA: Blackwell.

Lee, A., & Mok, P. (2021). Lexical Tone. In R.-A. Knight & J. Setter (Eds.), *The Cambridge Handbook of Phonetics* (pp. 185-208). Cambridge, UK: Cambridge University Press. doi:10.1017/9781108644198.008

Proctor, M. (2021). Consonants. In R.-A. Knight & J. Setter (Eds.), *The Cambridge Handbook of Phonetics* (pp. 65-105). Cambridge, UK: Cambridge University Press. doi:10.1017/9781108644198.004

Quijada, J. (2011). *A grammar of the Ithkuil language*.

Robinson, S. (2006). The Phoneme Inventory of the Aita Dialect of Rotokas. *Oceanic Linguistics*, *45*, 206-209. doi:10.1353/ol.2006.0018

Weckwerth, J. (2021). Vowels. In R.-A. Knight & J. Setter (Eds.), *The Cambridge Handbook of Phonetics* (pp. 40-64). Cambridge, UK: Cambridge University Press. doi:10.1017/9781108644198.003
